# Predictors of maternal and neonatal complications in women with severe valvular heart disease during pregnancy in Tunisia: a retrospective cohort study

**DOI:** 10.1186/s12884-021-04259-6

**Published:** 2021-12-08

**Authors:** Rania Hammami, Mohamed Ali Ibn Hadj, Yosra Mejdoub, Amine Bahloul, Selma Charfeddine, Leila Abid, Samir Kammoun, Abdallah Dammak, Kais Chaabene

**Affiliations:** 1 Department of Cardiology, Hedi Chaker Hospital, 3029 Sfax, Tunisia; 2 Research Unit UR 17ES37, Faculty of Medicine, University of SFAX, Sfax, Tunisia; 3 Department of Obstetrics & Gynecology, Hedi Chaker Hospital, Sfax, Tunisia; 4 Department of Epidemiology, Hedi Chaker Hospital, Sfax, Tunisia

**Keywords:** Pregnancy, Severe valvular heart disease, Neonatal complications, Prognosis

## Abstract

**Background:**

Severe valvular heart disease, especially stenosis, is a contraindication for conception according to the World Health Organization. This is still encountered in countries with a high rheumatic fever prevalence. The objective of this study was to determine predictors of maternal cardiac, obstetric and neonatal complications in pregnant women with severe valve disease.

**Methods:**

This is an observational retrospective cohort study of all pregnant women with severe heart valvulopathy who gave birth between 2010 and 2017.

**Results:**

We included 60 pregnancies in 54 women. Cardiac complications occurred during 37 pregnancies (61%). In multivariate analysis, parity (aOR =2.41, 95% CI[1.12–5.16]), revelation of valvulopathy during pregnancy (aOR = 6.34; 95% CI[1.26–31.77]), severe mitral stenosis (aOR = 6.98, 95% CI[1.14–41.05],) and systolic pulmonary arterial pressure (aOR =1.08, 95% CI[1.01–1.14]) were associated with cardiac complications. Obstetrical complications were noted during 19 pregnancies (31.8%). These complications were associated with nulliparity (aOR = 5.22; 95% CI[1.15–23.6]), multiple valve disease (aOR = 5.26, 95% CI[1.19–23.2]), systolic pulmonary arterial pressure (aOR =1.04, 95% CI[1.002–1.09]), and treatment with vitamin K antagonists (aOR = 8.71, 95% CI[1.98–38.2]). Neonatal complications were noted in 39.3% of newborns (*n* = 61) and these were associated with occurrence of obstetric complications (aOR = 16.47, 95% CI[3.2–84.3]) and revelation of valvulopathy during pregnancy (aOR = 7.33, 95% CI[1.4–36.1]).

**Conclusions:**

Revelation of valvular heart disease during pregnancy is a predictor of not only cardiac but also neonatal complications. Valvular heart disease screening during pre-conceptional counseling is thus crucial.

**Supplementary Information:**

The online version contains supplementary material available at 10.1186/s12884-021-04259-6.

## Background

Pregnancy is associated with several hemodynamic modifications which could disturb the function of the cardiac pump, especially in women with cardiac diseases. The prevalence of heart disease in pregnant women is estimated between 1 and 4% [1, 2]. According to the largest multicenter registry of heart diseases in pregnant women, “ROPAC”, the most prevalent cardiac diseases are rheumatic valvular pathology in low- and middle-income, and congenital heart diseases in high-income countries [3, 4]. The World Health Organization (WHO) considers severe stenotic valvular heart diseases as a contraindication for conception (WHO IV category) [5]. Because rheumatic heart disease is still prevalent in Tunisia [6], managing pregnant women with such conditions is a daily challenge for obstetricians, cardiologists, and anesthesiologists. Clinicians thus have to deal with two main challenges: 1. should they opt for termination of pregnancy due to the huge maternal and fetal risks? 2. what is the most appropriate mode of birth in case of continuation of pregnancy?

This study aimed at assessing both maternal and neonatal prognosis in pregnant women with at least one severe cardiac valve disease and identifying its risks.

## Methods

This is an observational retrospective cohort study that includes a cohort of all consecutive pregnant women with at least one severe cardiac valve disease during the period between January 2010 and December 2017, managed in both cardiology and obstetric departments of Hedi Chaker Hospital in Sfax, Tunisia. Pregnancies in women with cardiac valve prosthesis, as well as pregnancies in women who had either spontaneous or voluntary abortions were excluded. Baseline clinical, electrocardiographic and echocardiographic variables were collected from the medical files and included: maternal age, educational level, parity, cardiac lesions, prior cardiac interventions, New York Heart Association (NYHA) functional class, cardiac rhythm, ventricular systolic function, valvular function, and pulmonary arterial systolic pressure. Severity of valve lesions was assessed according to the guidelines of the European Society of Cardiology [7].

Women were called to get information about both contraception use after birth and eventual new pregnancies before treating the cardiac valve disease.

Women who refused to give verbal consent were excluded.

Maternal cardiac complications were defined as maternal cardiac death, new episode of arrhythmia requiring treatment, heart failure, thromboembolic events, clinical overdose with anticoagulants, endocarditis and hospitalization for cardiac reasons during pregnancy and up to 1 week postpartum.

Obstetric complications were defined as maternal death for obstetric causes up to 1 week postpartum, gestational diabetes, preeclampsia, threatening preterm birth, premature rupture of membranes and postpartum hemorrhage.

Neonatal complications were defined as preterm birth < 37 weeks, small-for-gestational-age (<10th centile), hospitalization in the neonatology department, and early neonatal death.

Categorical variables are presented as frequencies and percentages while differences between groups are assessed using χ2 tests. Continuous variables are presented as mean and standard deviation or as median and interquartile ranges as appropriate, and differences are assessed using Student’s t-test or Mann-Whitney U tests depending on data distribution. Univariate analysis to identify predictors of adverse events was performed using chi-square, Fisher exact or Student’s t-tests as appropriate. Univariate predictors of adverse events with *p* values < 0.2 were entered into a multivariate logistic regression model using backward elimination with a significant level of 0.05 and with adjusted odds ratios (aOR) with confidence intervals 95%.

## Results

Our cohort included 54 women with at least one severe valve disease and 60 pregnancies, leading to 59 spontaneous singleton births and one assisted twin birth (i.e. 61 newborns). During the study period, 80.624 births took place in our hospital. Six hundred thirty-nine cardiac ultrasound examinations were performed in pregnant women or during the first week after birth. The prevalence of valvular heart diseases was 6.7 per 10,000 births. Approximately 8.7 per 100.000 births had severe lesions.

Mean age of our cohort was 32.55 ± 5.6 years. More than half of the women were symptomatic of at least NYHA stage II dyspnea during the first trimester of pregnancy; however, 60% also showed anemia on their biological analysis. Baseline characteristics are shown in Table 1.

**Table 1:** Baseline characteristics of our population (54 women, 60 pregnancies, 61 Newborns)

Age (years)	Total number	32.55 ± 5.6 (range: 19–43)
Rural origin	54 women	29 women (53.7%)
Educational level	54 women	Analphabetic (3.7%),
		primary (59.2%),
		secondary (42.5%),
		university (5.5%)
Medical Antecedent	60 pregnancies	
Hypertension		5(8.3%)
Diabetes		2(3.3%)
Coronary diseases		1 (1.6%)
Idiopathic thrombocytopenia		1 (1.6%)
Parity		2.16 ± 1.15 (range 1–6)
Gravidity		2.8 ± 1.6 (range 1–5)
Obstetric Antecedent	60 pregnancies	43 (71.6%)
Previous CS		
One previous CS		12 (20%)
Two previous CS		4 (6.6%)
Miscarriage		
In Utero death		1 (1.6%)
Neonatal death		3 (5%)
Preeclampsia		4(6.6%)
Gestational diabetes		2(3.3%)
Dyspnea during the First Trimester		
	60 pregnancies	NYHA I: 30 (50%)
		NYHA II: 25 (41.6%)
		NYHA III: 5 (8.3%)
Diagnosis of valvular heart disease		
Before pregnancy	60 pregnancies	29 (48.3%)
During Pregnancy		28 (46.6%)
During labor		2 (3.3%)
During the first week		1 (1.6%)
Anemia during pregnancy	60 pregnancies	40 (66.6%)
MS	60 pregnancies	43 (71.6%)
AS		7 women (11.6%),
MR		6 women (10%),
MS + MR		1 patient (1.6%),
MS + AR		1 patient (1.6%),
PS		1 patient (1.6%)
Mean PASP (mmHg)	60 pregnancies	50.8 ± 16.03 (range: 20–95)
Anticoagulants in therapeutic dosage before or during pregnancy (%)	60 pregnancies	19 women (31.6%)
Mode of birth		
Cesarean	60 pregnancies	40 (66.6%)
Normal Vaginal birth		16 (26.6%)
Induced vaginal birth		3 (5%)
Gestational weeks at birth (median, min, max)	60 pregnancies	38 weeks (range:30–42)
Maternal Cardiac complications		37 (61.6%)
Heart failure	60 pregnancies	30 (50%)
Arrhythmia		2 (3.3%),
Embolic events		2 (3.3%),
Anticoagulants overdose		3 (5%)
Obstetrical complications	60 pregnancies	19 (31.6%)
Hemorrhage		3 (5%),
Preeclampsia		3(5%),
Gestational diabetes		2 (3.3%)
Threatening preterm birth		12 (20%),
Premature rupture of membranes		1 (1.6%)
Neonatal complications	61 Newborns (1 Twin)	24 (39.3%)
Small-for-gestational-age		12 (19.6%)
Preterm birth		9(14.7%)
Hospitalization		3(4.9%)

*MS* mitral stenosis, *A S* Aortic stenosis, *MR* mitral regurgitation, *AR* aortic regurgitation, *PS* pulmonary stenosis, *CS* Cesarean Section, *PASP* mean pulmonary arterial systolic pressure

Pregnancy had revealed valvular disease in 31 women (52%) and mean gestational age at diagnosis was 27 ± 6.7 weeks (range: 12–38 weeks).

Median gestational age of birth was 38 weeks (range: 30–42); preterm birth occurred in 12 pregnancies (20%) and there was only one case of > 42 weeks. This woman was not aware of her valvulopathy which was discovered during labor following acute pulmonary edema.

Pulmonary edema disclosed valve disease in 21 women during pregnancy. In nearly half pregnancy was unplanned (31 women). In this group, only 13/31 women (41.9%) used contraception at the time of conception (micro-progestatives in five women, intra-uterine device in one, and periodic abstinence in seven). The most prevalent valve disease was mitral stenosis (43/54, i.e. 79.6%). Percutaneous mitral commissurotomy was indicated in 19 parturient women out of 43. However, 11 of these women did not benefit from the procedure either because they refused it or because of a late, peripartum diagnosis of valvulopathy. Thus, this procedure was performed in only eight women with uncomplicated procedural courses. Among these women, there were seven vaginal births and one cesarean section following an obstetrical indication.

In 41 pregnancies (68%), birth occurred by cesarean section. In three pregnancies, vaginal birth occurred assisted with oxytocin was performed as valvulopathy was discovered post-partum. Indication for cesarean section was cardiac in 30 pregnancies (73%) and obstetrical in 11 pregnancies. Cesarean section was performed electively in most pregnancies (*n* = 36; 88%).

As far as newborns are concerned, median birth weight was 3000 g (range: 1500 g − 3750 g). Median Apgar score on the first minute was 9 (range: 3–9). Apgar score at 5 min was significantly lower in preterm than in full-term newborns: 7.16 ± 1.61 versus 8.66 ± 0.73, *p* = 0.01.

All newborns had an Apgar score ≥ 7 at 5 min, except for one who was born from a first twin pregnancy, whose score was = 3 at birth. He was hospitalized in neonatology for 17 days because of respiratory distress and the evolution was favorable.

In our population, 44 cardiac events occurred in 37 pregnancies (61.7%). Most cardiac events (*n* = 35, 79.5%) occurred antepartum, while the others occurred during labor or postpartum following discharge (*n* = 9, 20.5%) (Tables 2 and 5). The most common cardiac complications were heart failure (in 36 pregnancies) and arrhythmias (23 pregnancies). Only one woman, with a twin pregnancy, had cardiac arrest because of ventricular arrhythmia 2 h after mitral commissurotomy. Severe hypokalemia was diagnosed and the woman was transferred to the intensive care unit with favorable recovery. At gestational age of 30 weeks, birth of the two babies took place by cesarean section.

**Table 2:** Comparison of groups according to maternal cardiac complications

	MCC+(*n* = 37)	MCC-(*n* = 23)	*p*
Mean Age (SD)	33.4 ± 5.5	31.17 ± 5.4	0.13
Rural origin (%)	22(59.5)	23(30.4)	**0.029**
Low educational level (%)	26(70.3)	8(34.8)	**0.007**
Parity	2.45 ± 1.21	2.21 ± 1.16	**0.038**
Gravidity	3.16 ± 1.81	2.21 ± 1.16	**0.01**
Nulliparity (%)	11 (29.7)	10(43.5)	0.27
Unwanted pregnancy (%)	21(56.7)	9(39.1)	0.184
Unattended pregnancy (%)	29 (79.3)	20(87)	0.31
Hypertension before pregnancy (%)	3(8.1)	2(8.6)	0.64
Valve disease revealed during pregnancy (%)	23(62.2)	8(34.8)	0.039
NYHA≥2 (%)	21(56.8)	10(43.5)	0.31
Atrial fibrillation	18(48.6)	5(21.7)	**0.037**
Multiple valve disease (%)	19(51.4)	9(39.1)	0.35
Severe MS (%)	32(86.5)	11(47.8)	**0.001**
Severe AS (%)	5(13.5)	5(21.7)	0.31
Severe MR (%)	4(10.8)	6(26.1)	0.12
Severe AR (%)	1(2.7)	1(4.3)	0.62
LVEF (%)	57.4 ± 7.2	61.13 ± 2.2	0.011
PASP (mmHg)	57.29 ± 15.8	40.39 ± 9.7	**< 0.001**
LA diameter (mm)	52.72 ± 7.35	47.39 ± 6.04	**0.005**
Anemia ((%)	24 (64.9)	12 (52.2)	0.32

*AS* Aortic stenosis, *AR* Aortic regurgitation, *LVEF* Left ventricle ejection fraction, *LA* Left atrium, *MCC* maternal cardiac complications, *MS* Mitral stenosis, *MR* Mitral regurgitation, *NYHA* New York Heart Association, *PASP* Mean pulmonary arterial systolic pressure

Twenty-three obstetric events occurred in 19 pregnancies (31.8%) (Tables 3 and 4). Concerning the 61 newborns, 27 fetal events occurred in 24 newborns (39.3%) (Tables 5). On multivariate analysis predictors of neonatal complications were occurrence of obstetric events (aOR = 16.47, 95% CI [3.2–84.3]) and the revelation of valve disease during pregnancy (aOR = 7.33, 95% CI[1.4–36.1]) (Table 5). Overall, no maternal or neonatal death was observed. Three women showed preeclampsia and were treated with intravenous diuretics because of pulmonary edema.

**Table 3:** Comparison of groups according to obstetric complications

	OC+ (*n* = 19)	OC-(*n* = 41)	*P* value
Mean Age (SD)	34 ± 4.61	31 ± 5.87	0.15
Age > 35 years (%)	9(47.4)	14(34.1)	0.32
Rural Origin (%)	8(42.1)	21(51.2)	0.51
Parity	2.15 ± 1.3	2.17 ± 1.09	0.67
Nulliparity	9(47.4)	12(29.3)	0.17
Gravidity	2.84 ± 1.67	2.78 ± 1.66	0.88
Unwanted pregnancy (%)	10(52.6)	20(48.8)	0.78
Unattented pregnancy (%)	4(21.1)	7(17.1)	0.48
Hypertension (%)	2(10.5)	3(7.3)	0.51
Diabetes (%)	1(5.3)	1(2.4)	0.53
Pre-eclamptic Antecedent (%)	2(10.5)	2(4.9)	0.37
Previous Cesarean (%)	8(42.1)	8(19.5)	0.06
Previous Abortion (%)	5(26.3)	8(19.5)	0.39
Valve disease revealed during pregnancy (%)	7(36.8)	24(58.5)	0.11
NYHA≥2 (%)	10(52.6)	2(51.2)	0.91
Atrial fibrillation (%)	12(63.2)	11(26.8)	0.007
Maternal cardiac complications (%)	14(73.7)	23(56.1)	0.19
Multiple valve disease (%)	12(63.2)	15(39)	0.08
Severe MS (%)	15(78.9)	28(68.3)	0.39
Severe AS (%)	3(15.8)	7(17.1)	0.6
Severe MR (%)	3(15.8)	7(17.1)	0.609
Severe AR (%)	1(5.3)	1(2.4)	0.53
LVEF (%)	60.26 ± 3.1	58.19 ± 6.98	0.42
SAPS (mmHg)	57.1 ± 16.9	47.9 ± 14.9	0.03
LA diameter (mm)	53.5 ± 8.02	49.4 ± 6.7	0.05
Anemia (%)	11(57.9)	25(61)	0.82
Anticoagulant use (%)	11(57.9)	8(19.5)	0.003
Diuretic use (%)	12(53.6)	15 (80)	0.08
Beta-blockers use (%)	6(40)	4(14.3)	0.057
Cardiac events (%)	14(73.7)	23(56.1)	0.19

*AS* Aortic stenosis, *AR* Aortic regurgitation, *LVEF* Left ventricle ejection fraction, *LA* Left atrium, *MS* Mitral stenosis, *MR* Mitral regurgitation, *NYHA* New York Heart Association, *PASP* Mean pulmonary arterial systolic pressure, *OC* obstetric complications

**Table 4:** Comparison of groups according to neonatal complications

	NC+ (*n* = 24)	NC-(*n* = 37)	*P* value
Age (years)	32.45 ± 4.61	32.54 ± 6.09	0.95
Rural origin (%)	12(50)	17(45.9)	0.75
Low educational level (%)	13(54.2)	22(59.5)	0.68
Parity	2.29 ± 1.04	2.08 ± 1.21	0.3*
Gravidity	3.04 ± 1.65	2.59 ± 1.65	0.24*
Unwanted pregnancy (%)	13(54.2)	17(45.9)	0.53
Unfollowed pregnancy (%)	4(16.7)	7(18.9)	1**
Hypertension before pregnancy (%)	3(12.5)	2(5.4)	0.37**
Valve disease revealed during pregnancy (%)	16(66.7)	16(43.2)	0.07
NYHA≥2 (%)	14(58.3)	17(45.9)	0.34
Atrial fibrillation (%)	12(50)	11(29.7)	0.11
Maternal cardiac events (%)	20(83.3)	18(48.61)	0.006
Multiple valve disease (%)	11(45.8)	17(45.9)	0.99
Severe MS (%)	19(79.2)	25(67.6)	0.32
Severe AS (%)	3(12.5)	7(18.9)	0.7**
Severe MR (%)	4(16.7)	6(16.2)	1**
Severe AR (%)	1(4.1)	1(2.7)	1**
LVEF (%)	57.95 ± 8.49	59.5 ± 3.79	0.7*
PASP (mmHg)	58.6 ± 16.1	45 ± 13.8	0.003*
LA diameter (mm)	53.1 ± 8.3	49.29 ± 6.2	0.04
Anemia (%)	16(66.7)	21(56.8)	0.43
Hemoglobin (g/dl)	10.15 ± 1.22	10.36 ± 1.24	0.37*
Anticoagulant use (%)	9(37.5)	10(27)	0.38
Cardiac events (%)	20(83.3)	18(48.6)	0.006
Obstetric events (%)	14 (58.3)	6(16.2)	0.001

*AS* Aortic stenosis, *AR* Aortic regurgitation, *LVEF* Left ventricle ejection fraction, *LA* Left atrium, *MS* Mitral stenosis, *MR* Mitral regurgitation, *NC* neonatal complications, *NYHA* New York Heart Association, *PASP* Mean pulmonary arterial systolic pressure

*test U-Mann Whitney

**Fisher test

**Table 5:** Predictors of maternal, obstetric and neonatal cardiac complications on univariate and multivariate regression

	Univariate regression		Multivariate regression	
Predictors of maternal complications				
	OR	CI 95%	aOR	CI 95%
Parity	2.09	[1.14–3.82]	2.41	[1.12–5.16]
Valve disease revealed during pregnancy	3.08	[1.04–9.11]	6.34	[1.26–31.77]
Severe Mitral stenosis	6.98	[2–24.32]	6.84	[1.14–41.05]
PASP (mmHg)	1.1	[1.04–1.16]	1.08	[1.01–1.14]
Predictors of obstetric complications				
Nulliparity	2.17	[0.7–6.69]	5.22	[1.15–23.6]
Multiple valve disease	2.67	[0.87–8.24]	5.26	[1.19–23.2]
PASP (mmHg)	1.03	[1.001–1.076]	1.04	[1.002–1.09]
Anticoagulant use	5.67	[1.71–18.7]	8.71	[1.98–38.2]
Predictors of neonatal complications				
Obstetric events	7.2	[2.1–23.8]	16.47	[3.2–84.3]
Valve disease revealed during pregnancy	2.6	[0.9–7.6]	7.33	[1.4–36.1]

*aOR* adjusted Odds Ratio, *CI* confidence Interval, *OR* Odds Ratio, *PASP* pulmonary arterial systolic pressure

Pregnancies with cardiac complications showed significantly more fetal events (51.4% versus 17.4%, *p* = 0.009) but no differences were observed in obstetric complications (37.8% versus 21.7%, *p* = 0.19).

The rate of preterm birth was 20% in our series (in 12 pregnancies). Two women had spontaneous preterm birth, while 10 had iatrogenic preterm births, nine of whom (9/12 = 75%) had severe mitral stenosis. Pulmonary arterial pressure was significantly higher in those with preterm birth (60.08 ± 16.81 versus 48.08 ± 15.94 mmHg, *p* = 0.04). After 1 year, the index pregnancy, only 31 women used contraception (31/60 pregnancies = 51.7%). Among the other women, 6 (10%) had a new pregnancy even before treating their severe valve heart disease.

## Discussion

Our cohort analyzed the outcomes of pregnancies in women with severe heart valve disease, cardiac events occurred approximately in two women out of three, obstetric events occurred approximately in one women out three and neonatal events occurred in approximately 4 newborns out of 10. The revelation of heart valve disease during pregnancy was a common predictor of both cardiac and obstetric eventsCardiovascular diseases are the most common causes of indirect maternal death during pregnancy and childbirth up to 6 weeks postpartum [2]. Knowledge of the risks associated with such conditions in pregnant women and their management is of pivotal importance. However, the number of published cases is limited and most recommendations are based on expert consensus with no strong level of evidence [8].

Tolerance of severe valvular heart diseases during pregnancy is little recognized since the prevalence of rheumatoid arthritis is decreasing. Nowadays, more and more women are getting pregnant at an older age, thus, the prevalence of pregnant women with severe cardiac valve disease remains high in low and middle income countries. The current study was carried out to evaluate the maternofetal outcomes in pregnant women with severe valve lesions. The recently published Global Rheumatic Heart Disease Registry (REMEDY) included 3.343 women with rheumatic heart disease in 12 African countries, India and Yemen; median age was 28 years [9]. The majority (2137;63.9%) had moderate to severe valvulopathy which was complicated by congestive heart failure in (705; 33.4%), pulmonary hypertension in (599;28.8%), atrial fibrillation in (449;21.8%), stroke in(151;7.1%), infectious endocarditis in (86;4%), and major bleeding in (57;2.7%). In 1.825 women of reproductive age (aged 12–51), only (65; 3.6%) used contraception. In a Tunisian study analyzing the epidemiological profile of cardiac women who gave birth in a Tunis maternity center between January 2010 and December 2012, out of 19.655 deliveries studied, the prevalence of heart disease was 1 in 351 deliveries (0.285%; i.e., 56 women). Mean age was 30.89 ± 5.3 years (ranges between 21 and 42 years). Out of the 56 cases of cardiac women, 35 women (62.5%) had valvulopathy, which translates into a prevalence of 0.17% [10]. In our cohort, the mean age was 32.5 ± 5.6 years and the prevalence of heart valve disease was about 0.49% while that of severe disease was estimated at 0.074%.

Cardiac complications are frequent in pregnant women with cardiac valve diseases and vary between 13 and 35.6% according to previous studies [11–15] (Table 6). Certainly, the rate of complications should be higher in women with severe lesions, especially when the condition is discovered only during pregnancy. In the CAPREG II study, the overall maternal cardiac event rate during pregnancy for mWHO I (no increased risk), mWHO II (small increased risk), mWHO III (significantly increased risk), and mWHO IV (extremely high risk) was 3.1, 12.8, 21.1, and 35.6%, respectively. In our cohort, two pregnancies out of three showed cardiac events. Pulmonary edema seems to be the most prevalent complication; nevertheless, the advancement of therapeutic strategies improved the prognosis. Maternal cardiac death became rare; in the recent CAPREG II study, it occurred in only 11 pregnancies (0.6%) [12]. No cardiac death occurred during the practice of a tertiary care center including women with pulmonary hypertension, [16]. In our series, no maternal death was noted; only one case of cardiac arrest occurred because of hypokaliemia, and the recovery was obtained. However, cardiac mortality remains much higher than that in the obstetrical population [17–20].

**Table 6:** Predictors of cardiac, obstetric, and fetal events in literature

	CAPREG I (8)	ROPAC REGISTRY [3]	CAPREG II [12]	Our Study
Study Period	1994–1999	2008–2014	2001–2014	2010–2017
Publication year	2001	2016	2018	–
Methodology	Prospective (Canadian registry)	Prospective (multicenter European Registry)	Prospective cohort (Canadian registry)	Retrospective cohort
Population	*N* = 599 (congenital and acquired disease)	*N* = 2742,VHD = 865	*N* = 1938	*N* = 60 pregnancies in 54 women with severe valve disease, 61 newborns
Age (years)	28 ± 6	29.2 ± 5.5	30.6 ± 5.6	32.5 ± 5.6
MCC incidence	13%	20.6%	16%	61%
Independent Predictors of MCC	*Cardiac disease antecedent *NYHA≥2 /cyanosis *Left heart obstruction *LVEF ≤40%	*Pre-pregnancy signs of heart failure *in advanced countries. Atrial fibrillation and no previous cardiac intervention	*Prior cardiac events or arrhythmias. *poor functional class or cyanosis. *high-risk valve disease/left ventricular outflow tract obstruction. *systemic ventricular dysfunction. *no prior cardiac interventions. *Mechanical valves. *high-risk aortopathies. *pulmonary hypertension. *late pregnancy assessement	*Parity *Revelation of the valve disease by pregnancy *Mitral stenosis *Systolic pulmonary hypertension
OC incidence	7%	8.4%	–	31%
Independent Predictors of OC	*Primiparity *Aortic coarctation. *lupus *Anticoagulant use. *cyanosis	*Primiparity. *hypertension before pregnancy	–	*Nulliparity *PASP *Anticoagulant use *Multiple valve disease
NC incidence	20%	23.7%	–	39.3%
Independent predictors of NC	Multigestity NYHA II or cyanosis Anticoagulant use Tobacco use Left heart obstruction	Multigestity Anticoagulant use Pregnancy in underdeveloped countries	–	Maternal cardiac complications Valve disease revealed by pregnancy

*LVEF* Left Ventricle Ejection Fraction, *MCC* maternal cardiac complications, *NC* Neonatal complications, *NYHA* New York Heart Association, *OC* obstetrical Complications, *VHD* valve heart disease

Several studies tried not only to determine the predictors of cardiac events but also to analyze them in order to validate scores.

The multicentric CAPREG (Cardiac Disease in Pregnancy Study) including women both with congenital and acquired diseases, was the first to develop CAPREG Score. Predictors of cardiac events were: left heart obstruction, cyanosis or dyspnea before pregnancy, cardiac antecedents and systolic LV dysfunction [14, 21]. In the European Registry of Pregnancy and Heart Disease (ROPAC), including 2.966 women, valvular heart diseases account for 25% of pregnant women with cardiovascular disease. Mitral valve diseases – both stenosis and regurgitation – were the most common valvular lesions (63%), followed by aortic valve disease (23%) (Table 6). In this registry, signs of heart failure before pregnancy, atrial fibrillation and no previous cardiac intervention were strong predictors of cardiac events. In our cohort, cardiac complications occurred especially in stenotic lesions; regurgitant lesions were well-tolerated and did not affect the possibility of carrying a pregnancy to full term. In stenotic lesions, increased cardiac output causes a significant rise in the transvalvular gradient of 50%, mainly between the first and second trimesters [22]. That is why congestive heart failure occurs more commonly in the third trimester or early postpartum period, whereas most arrhythmias occur in the antenatal period.

Mitral valve stenosis was usually not tolerated and was found to be an independent predictor of cardiac complications. In fact, the pregnancy period is characterized by an increase of cardiac output brought by increased heart rate and stroke volume, and oppositely, a decrease of peripheral resistance by peripheral vasodilatation. The drop of vascular resistance explains the tolerance of regurgitant lesions [6, 23–25].

Pregnancy outcomes in two large centers in the United States reported a risk of pulmonary edema occurrence in pregnant women with mild, moderate and severe mitral stenosis, between 11 and 24%, 34 and 61%, and 56 and 78%. The rate of occurrence of atrial fibrillation varied between 0 and 7% in the case of mild stenosis, 10 and 22% in the case of moderate stenosis, and between 22 and 33% in the case of severe lesions [15, 26].

Percutaneous mitral dilation seems to remarkably improve the hemodynamic condition of these women to an extent where even a vaginal delivery is made possible in most cases. According to the recent ESC guidelines, intervention should be considered before pregnancy in women with MS and valve area < 1.5 cm^2^ and should be considered in pregnant women with severe symptoms or systolic pulmonary artery pressure > 50 mmHg despite medical therapy [8].

In our study, the revelation of cardiac valve disease during pregnancy was also found to be a strong predictor not only of cardiac complications but also of neonatal complications. This result accords with other predictors found in the ROPAC registry and the CAPREG II study which are lack of intervention before pregnancy as well as delayed pregnancy assessment. Therefore, it is strongly recommended to assess pre-pregnancy risk and to counsel all women with known or suspected congenital or acquired heart valve diseases and aortic disease [27]. It is also recommended to perform risk assessment in all women of childbearing age with cardiac diseases before, as well as after conception, using the mWHO classification of maternal risk [27]. Generally, women who receive late pregnancy assessment have more frequent adverse cardiac outcomes during pregnancy, which may be attributed to delayed access to appropriate risk stratification, follow-up, and management plan.

However, we noted that in our practice, even though the rate of illiteracy among women was low (3%), use of contraception is infrequent. In our cohort, six women showed cardiac complications and became pregnant again before treating the heart valve diseases. The cardiologist plays a pivotal role in the therapeutic education of these women and the prescription of adequate contraception while discussing with the gynecologist.

The obstetrical risks remain poorly described in the literature. The main parameter to be evaluated is the mode of birth, which depends, in this context, on the maternal tolerance of pushing efforts and the possibility of epidural analgesia. According to the recent guidelines, a birth plan should be made between gestational age of 20–30 weeks, detailing of pregnancy detailing induction, management of labor, birth, and post-partum follow-up; Moreover, the induction of labor should be considered at 40 weeks of gestation in all women with cardiac disease [8].

Obstetric complications were 3 to 4 times more common in our population compared to the ROPAC and CAPREG studies (Table 6). Low parity (nulliparous or primiparous) is a predictor of obstetrical events in many studies, especially that it increases the risk of preeclampsia [28] (Table 6).

In our cohort, preterm labor threat was the most common complication. This was mainly due both to uterine muscle hypoxia and to acute heart failure, which were common occurrences in our women.

Pre-eclampsia was more common in women with aortic valve disease and left ventricle dysfunction because of low cardiac output and, as expected, in nulliparous women and those with pre-existing hypertension. In our series, all the cases of preeclampsia occurred in women treated with intravenous diuretics because of acute heart failure. This is a frequently faced challenge. The use of diuretics results in placenta hypoperfusion which is the main mechanism of preeclampsia; therefore, diuretics should be avoided in such women. The use of anticoagulants is also a predictor of obstetrical complications, mainly hemorrhage. In our cohort, this factor increased the risk of obstetrical events by 8 times. Indeed, the association between hemorrhage and anemia, frequently diagnosed in our women, worsens the prognosis and results in heart decompensation. It is therefore recommended to anticipate the timing of birth to ensure a safe and effective peripartum anticoagulation.

Neonatal prognosis is closely correlated with maternal prognosis. Anyway, in our series, we found the occurrence of obstetric complications (aOR = 16.47, 95% CI[3.2–84.3]) and the revelation of valvulopathy during pregnancy (aOR = 7.33, 95% CI[1.4–36.1]) to be strong predictors of neonatal complications. In fact, the delayed discovery of valvulopathy may expose the fetus to a longer duration of hemodynamic stress, especially with a risk of placental hypoperfusion, hypotrophy, and prematurity. The immunological status of these newborns will also be precarious with a higher risk of hospitalization in the neonatal care unit. In the ROPAC registry, the rate of fetal complications among women with WHO IV heart disease was 31%, which is comparable to the 40% rate in our series. Predictors of these complications in ROPAC were multigravidity, treatment with anticoagulants, diabetes and life in low income countries (Table 6).

Symptomatic women (NYHA class III-IV), and oral anticoagulants, were the main reasons for induced preterm birth. In the case of spontaneous prematurity, both inflammatory processes and utero-placental ischemia can initiate preterm labor [29]. Certainly, maternal hypoxia increases cytokines and oxygen free radicals, which may cause abnormal placentation; these conditions occurred mostly in women with congenital heart disease [30]. In our cohort, most cases of preterm birth were induced, which is why pulmonary systolic pressure was significantly higher in mothers of preterm babies. In a Japanese cohort including 857 women with cardiac disease, ischemic cardiac disease (48.3%), and valvular heart disease (44.3%) were related to the highest risk of induced prematurity. The rate of cesarean section was 81.7 and 68.8%. In our series, we noted only 20% of preterm birth while previous studies (3–6) showed that both induced and spontaneous preterm births were less common in low income than in high income countries. This was explained by the advanced age with uterine dysfunction for spontaneous preterm birth and quicker access to obstetric care facilities in high income countries.

### Study limitations

The main limitations of our study involved the retrospective design, on the one hand, and the small sample size on the other hand. These two limitations could be explained by the low prevalence of severe valve diseases among pregnant women as they contra-indicate conception. Moreover, we included only term-pregnancies since abortions and non-viable pregnancies are multifactorial which made us unable to conclude that there is a relationship between these obstetric events and the severity of valvulopathies.

## Conclusions

To conclude, our cohort demonstrated that women with severe heart valve diseases should be counseled about pregnancy risks and fetal problems. It is crucial to early detect such cardiac lesions during premarital consultation through a meticulous interrogation and cardiac auscultation.

Since mitral stenosis is the most commonly encountered lesion, percutaneous treatment should be attempted whenever possible and performed by an experimented operator. This improves hemodynamic conditions and offers more chances of vaginal birth.

Moreover, obstetricians should give priority to vaginal delivery – with triggering if necessary, use epidural analgesia, if not contraindicated, and shorten birthing time, especially in women treated with anticoagulants.

Finally, in low and middle income countries, much more effort should be given to the organization of contraception campaigns in cardiac women, which necessitates close collaboration between both cardiologist and obstetrician.

## Supplementary Information

**Additional file 1.**

## Data Availability

All data generated or analyzed during this study are included in this published article and its supplementary information files.

## Abbreviations

AR: Aortic regurgitation
AS: Aortic Stenosis
CS: Cesarean
CI: Confidence Interval
LA: Left Atrium
LVEF: Left ventricle ejection fraction
MCC: Maternal cardiac complications
MR: Mitral regurgitation
MS: Mitral stenosis
NYHA: New York Heart Association
NC: Neonatal complications
aOR: Adjusted Odds ratio
OC: Obstetric complications
PS: Pulmonary stenosis
PASP: Mean pulmonary arterial systolic pressure
WHO: World Health Organization
