# Supplementary material for: Predictors of maternal and neonatal complications in women with severe valvular heart disease during pregnancy in Tunisia: a retrospective cohort study
Source: BMC Pregnancy Childbirth. 2021 Dec 8;21:813. doi: 10.1186/s12884-021-04259-6 (PMC8653539; doi:10.1186/s12884-021-04259-6)
Supplement: Supplementary file 1 — Additional file 1. [file 12884_2021_4259_MOESM1_ESM.pdf]

numerofic	datenaissa	niveauinte	profession	origine	anneegros	tranche_ag	age_grosse	gross_prog
1	14-juil-72	2,00	faf	gabes	2010	5,00	38,00	2,00
11	########	2,00	faf	kerkna	2015	5,00	39,00	1,00
61	########	2,00	faf	sfax	2012	4,00	35,00	2,00
2	########	3,00	faf	gabes	2013	4,00	31,00	2,00
65	13-oct-87	2,00	faf	gabes	2017	3,00	30,00	1,00
3	########	2,00	faf		2014	3,00	29,00	1,00
4	16-juil-81	2,00	faf	mahres	2016	4,00	35,00	2,00
6	########	2,00	faf	sfax	2011	2,00	25,00	1,00
52	03-oct-87	3,00	faf	sfax	2013	3,00	26,00	2,00
7	29-juin-88	3,00	faf	rgueb	2013	2,00	25,00	1,00
46	########	4,00	professeur	sidi bouzid	2017	5,00	37,00	2,00
8	########	2,00	faf	sidibouzid	2013	5,00	39,00	2,00
45	15-oct-86	3,00	faf	sfax	2017	4,00	31,00	1,00
9	########	1,00	faf		2010	6,00	41,00	2,00
10	27-oct-85	3,00	faf	sfax	2011	4,00	26,00	2,00
12	31-oct-86	3,00	faf	Jerba	2015	3,00	29,00	1,00
60	########	4,00	étudiante	sfax	2013	1,00	20,00	1,00
41	########	2,00	faf	jbeniena	2013	6,00	42,00	2,00
49	########	2,00	faf	mednine	2015	3,00	28,00	1,00
59	########	3,00	professeur	sfax	2017	4,00	33,00	2,00
64	07-juil-74	3,00	faf	sidibouzid	2017	6,00	43,00	2,00
14	########	2,00	faf	hencha	2015	5,00	36,00	2,00
15	########	2,00	faf	sidibouzid	2013	5,00	36,00	1,00
16	########	3,00	fonctionna	sfax	2010	3,00	30,00	1,00
17	########	3,00	fonctionna	sfax	2014	4,00	34,00	1,00
18	05-mai-82	2,00	faf	jbeniana	2016	4,00	34,00	2,00
19	########	3,00	faf	sfax	2015	4,00	33,00	2,00
54	25-oct-80	3,00	faf	kasserine	2012	4,00	32,00	1,00
20	12-mai-73	2,00	faf		2012	3,00	29,00	1,00
21	########	2,00	faf	hencha	2013	6,00	43,00	2,00
62	########	2,00	faf	sfax	2009	5,00	37,00	2,00
22	########	2,00	faf	sfax	2012	4,00	35,00	2,00
23	01-juin-93	2,00	faf	gabes	2012	1,00	19,00	1,00
55	#NULL!	2,00	patisiere		2017	5,00	36,00	2,00
24	27-nov-82	2,00	faf	jbeniena	2010	3,00	27,00	1,00
25	27-nov-82	2,00	faf	jbeniena	2015	4,00	32,00	2,00
56	########	3,00	faf	medenine	2016	5,00	40,00	1,00
26	03-oct-78	2,00	faf	mahres	2012	4,00	34,00	1,00
27	03-oct-78	2,00	faf	mahres	2015	5,00	37,00	1,00
29	03-oct-78	2,00	faf	mahres	2016	5,00	38,00	2,00
28	17-mai-84	3,00	ouvriere		2014	3,00	30,00	1,00
51	21-oct-81	4,00	professeur	sfax	2015	4,00	34,00	1,00
30	########	2,00	faf	hencha	2012	3,00	30,00	2,00
31	#NULL!	2,00	ouvriere		2013	4,00	34,00	1,00
32	########	2,00	faf	ben guerde	2017	6,00	41,00	1,00
48	30-avr-81	2,00	faf	sidi bouzid	2016	4,00	34,00	2,00
33	########	2,00	faf		2010	2,00	23,00	1,00
35	31-déc-88	3,00	faf		2016	3,00	27,00	1,00
37	25-juin-88	3,00	faf	sfax	2012	2,00	23,00	2,00

36	########	2,00	faf	skhira	2017	3,00	29,00	2,00
63	########	1,00	faf	sidibouzid	2017	4,00	34,00	1,00
38	########	2,00	faf	sfax	2015	4,00	35,00	2,00
39	########	3,00	faf	sfax	2013	4,00	32,00	2,00
53	########	2,00	faf	mednine	2016	5,00	36,00	1,00
58	########	3,00	faf	sfax	2017	2,00	23,00	2,00
40	########	3,00	faf	kasserinr	2015	5,00	36,00	1,00
57	########	3,00	ouvriere	medecine	2016	3,00	30,00	1,00
50	17-déc-83	3,00	secretaire	sfax	2012	3,00	29,00	2,00
43	########	3,00	faf	zarzis	2014	5,00	37,00	2,00
44	########	3,00	faf	sfax	2012	4,00	32,00	1,00

moyencon	gestite	parité	nbre_EV	uteruscicat	ATCD_avor	ATCD_MFI	Mortneona	causemort
calendrier	4,00	3,00	3,00	#NUL!	2,00	2,00	2,00	
	4,00	2,00	1,00	1,00	1,00	2,00	2,00	
auccun	4,00	3,00	2,00	0,00	1,00	2,00	2,00	
auccune	3,00	3,00	2,00	#NUL!	2,00	2,00	2,00	
	1,00	2,00	0,00	0,00	2,00	2,00	2,00	
	2,00	2,00	1,00	#NUL!	2,00	2,00	2,00	
calendrier	5,00	4,00	3,00	1,00	1,00	2,00	2,00	
	1,00	1,00	0,00	2,00	2,00	2,00	2,00	
microval	4,00	3,00	2,00	0,00	1,00	2,00	2,00	
	3,00	3,00	2,00	#NUL!	2,00	2,00	2,00	
calendrier	3,00	2,00	0,00	#NUL!	1,00	2,00	2,00	
aucun	5,00	5,00	4,00	#NUL!	2,00	2,00	2,00	
	2,00	2,00	1,00	0,00	2,00	2,00	2,00	
	3,00	2,00	2,00	#NUL!	2,00	2,00	2,00	
auccun	4,00	3,00	2,00	0,00	2,00	2,00	2,00	
	3,00	3,00	1,00	#NUL!	2,00	2,00	2,00	
auccun	1,00	1,00	0,00	0,00	2,00	2,00	2,00	
auccun	9,00	6,00	5,00	1,00	1,00	2,00	1,00	
	1,00	1,00	0,00	0,00	2,00	2,00	2,00	
calendrier	3,00	2,00	2,00	0,00	2,00	2,00	2,00	
calendrier	3,00	2,00	2,00	#NUL!	2,00	2,00	2,00	
	4,00	3,00	2,00	#NUL!	1,00	2,00	2,00	
	2,00	1,00	1,00	#NUL!	1,00	2,00	2,00	
	1,00	1,00	0,00	0,00	2,00	2,00	2,00	
	2,00	2,00	1,00	0,00	2,00	2,00	2,00	
auccune	4,00	3,00	3,00	1,00	2,00	2,00	2,00	
calendrier	3,00	2,00	1,00	#NUL!	2,00	1,00	2,00	
	3,00	3,00	3,00	0,00	2,00	2,00	2,00	
	1,00	1,00	0,00	#NUL!	2,00	2,00	2,00	
auccun	4,00	3,00	3,00	0,00	2,00	2,00	2,00	
calendrier	3,00	3,00	2,00	0,00	2,00	2,00	2,00	
aucun	7,00	5,00	5,00	1,00	2,00	2,00	1,00	inconnu
auccun	1,00	1,00	0,00	0,00	2,00	2,00	2,00	
	2,00	1,00	0,00	#NUL!	2,00	2,00	2,00	
auccun	2,00	1,00	1,00	0,00	2,00	2,00	2,00	
calendrier	3,00	2,00	2,00	0,00	2,00	2,00	2,00	
auccun	1,00	1,00	0,00	0,00	2,00	2,00	2,00	
	1,00	1,00	0,00	0,00	2,00	2,00	2,00	
	2,00	2,00	1,00	1,00	2,00	2,00	2,00	
auccun	3,00	2,00	2,00	2,00	2,00	2,00	2,00	
	2,00	1,00	0,00	#NUL!	1,00	2,00	2,00	
auccune	1,00	1,00	0,00	#NUL!	2,00	2,00	2,00	
auccune	4,00	3,00	2,00	#NUL!	2,00	2,00	1,00	
	2,00	2,00	1,00	#NUL!	2,00	2,00	2,00	
auccun	3,00	1,00	0,00	#NUL!	2,00	2,00	2,00	
auccun	2,00	2,00	2,00	0,00	2,00	2,00	2,00	
	1,00	1,00	0,00	#NUL!	2,00	2,00	2,00	
	1,00	1,00	0,00	#NUL!	2,00	2,00	2,00	
auccun	6,00	3,00	3,00	0,00	1,00	2,00	2,00	

auccune	4,00	3,00	2,00	#NUL!	2,00	2,00	2,00
	5,00	3,00	2,00	0,00	1,00	2,00	2,00
auccun	4,00	3,00	3,00	0,00	2,00	2,00	2,00
auccun	2,00	2,00	1,00	0,00	2,00	2,00	2,00
microprog	3,00	2,00	2,00	0,00	2,00	2,00	2,00
auccun	2,00	1,00	0,00	#NUL!	1,00	2,00	2,00
	1,00	1,00	0,00	#NUL!	2,00	2,00	2,00
aucun	1,00	1,00	0,00	0,00	2,00	2,00	2,00
calendrier	1,00	1,00	0,00	#NUL!	2,00	2,00	2,00
auccun	5,00	4,00	3,00	0,00	1,00	2,00	2,00
aucun	1,00	1,00	0,00	0,00	2,00	2,00	2,00

ATCD_Toxo	Sterilite	diabete	HTA	tabac	ATCD_DMI	atcd_meac	annee_dm	annee_dm
1,00	3,00	2,00	2,00	2,00	1,00	#NUL!	1999	2010
2,00	2,00	2,00	2,00	2,00	1,00	#NUL!	1994	1997
2,00	3,00	2,00	2,00	2,00	2,00	#NUL!	#NUL!	#NUL!
2,00	3,00	2,00	2,00	2,00	2,00	#NUL!	#NUL!	#NUL!
2,00	1,00	2,00	2,00	2,00	2,00	#NUL!	#NUL!	#NUL!
2,00	3,00	2,00	2,00	2,00	2,00	#NUL!	#NUL!	#NUL!
2,00	3,00	22,00	2,00	2,00	2,00	1,00	#NUL!	#NUL!
2,00	3,00	2,00	2,00	2,00	2,00	#NUL!	#NUL!	#NUL!
2,00	3,00	2,00	2,00	2,00	1,00	#NUL!	2009	#NUL!
2,00	3,00	2,00	2,00	2,00	1,00	#NUL!	2012	#NUL!
2,00	3,00	2,00	2,00	2,00	2,00	#NUL!	#NUL!	#NUL!
1,00	3,00	2,00	2,00	2,00	2,00	#NUL!	#NUL!	#NUL!
2,00	3,00	2,00	2,00	2,00	2,00	#NUL!	#NUL!	#NUL!
2,00	3,00	2,00	2,00	2,00	2,00	#NUL!	#NUL!	#NUL!
2,00	3,00	2,00	2,00	2,00	2,00	#NUL!	#NUL!	#NUL!
1,00	3,00	2,00	1,00	2,00	2,00	#NUL!	#NUL!	#NUL!
2,00	3,00	2,00	2,00	2,00	2,00	#NUL!	#NUL!	#NUL!
2,00	3,00	2,00	2,00	2,00	2,00	#NUL!	#NUL!	#NUL!
2,00	3,00	2,00	2,00	2,00	2,00	#NUL!	#NUL!	#NUL!
2,00	3,00	2,00	2,00	2,00	1,00	#NUL!	2010	#NUL!
2,00	3,00	2,00	2,00	2,00	1,00	#NUL!	2007	#NUL!
2,00	3,00	2,00	2,00	2,00	2,00	#NUL!	#NUL!	#NUL!
2,00	3,00	2,00	2,00	2,00	1,00	#NUL!	2008	#NUL!
2,00	3,00	2,00	2,00	2,00	2,00	#NUL!	#NUL!	#NUL!
2,00	2,00	2,00	2,00	2,00	2,00	2,00	#NUL!	#NUL!
2,00	3,00	2,00	2,00	2,00	2,00	#NUL!	#NUL!	#NUL!
2,00	3,00	2,00	2,00	2,00	2,00	#NUL!	#NUL!	#NUL!
2,00	3,00	2,00	1,00	2,00	2,00	#NUL!	#NUL!	#NUL!
2,00	3,00	2,00	2,00	2,00	2,00	#NUL!	#NUL!	#NUL!
2,00	3,00	2,00	2,00	2,00	22,00	#NUL!	#NUL!	#NUL!
2,00	3,00	2,00	2,00	2,00	2,00	#NUL!	#NUL!	#NUL!
1,00	3,00	2,00	2,00	2,00	1,00	#NUL!	2003	#NUL!
2,00	3,00	2,00	2,00	2,00	2,00	#NUL!	#NUL!	#NUL!
2,00	2,00	2,00	2,00	2,00	2,00	#NUL!	#NUL!	#NUL!
2,00	3,00	2,00	2,00	2,00	2,00	#NUL!	#NUL!	#NUL!
2,00	3,00	2,00	2,00	2,00	1,00	#NUL!	2010	#NUL!
2,00	3,00	2,00	2,00	2,00	2,00	#NUL!	#NUL!	#NUL!
2,00	3,00	2,00	1,00	2,00	1,00	#NUL!	2010	#NUL!
2,00	3,00	2,00	1,00	2,00	1,00	#NUL!	1996	2000
2,00	3,00	2,00	1,00	2,00	1,00	#NUL!	2010	#NUL!
2,00	3,00	2,00	2,00	2,00	2,00	#NUL!	#NUL!	#NUL!
2,00	3,00	2,00	2,00	2,00	2,00	#NUL!	#NUL!	#NUL!
2,00	3,00	2,00	2,00	2,00	2,00	#NUL!	#NUL!	#NUL!
2,00	3,00	1,00	2,00	2,00	2,00	#NUL!	2005	#NUL!
2,00	1,00	2,00	2,00	2,00	2,00	#NUL!	#NUL!	#NUL!
2,00	3,00	2,00	2,00	2,00	2,00	#NUL!	#NUL!	#NUL!
2,00	3,00	2,00	2,00	2,00	2,00	#NUL!	#NUL!	#NUL!
2,00	3,00	2,00	2,00	2,00	2,00	#NUL!	#NUL!	#NUL!
2,00	3,00	2,00	2,00	2,00	2,00	#NUL!	#NUL!	#NUL!

2,00	3,00	2,00	2,00	2,00	1,00	#NUL!	2013	#NUL!
2,00	2,00	2,00	2,00	2,00	2,00	#NUL!	#NUL!	#NUL!
2,00	3,00	2,00	2,00	2,00	2,00	#NUL!	#NUL!	#NUL!
2,00	3,00	2,00	2,00	2,00	2,00	#NUL!	#NUL!	#NUL!
2,00	3,00	2,00	2,00	2,00	2,00	#NUL!	#NUL!	#NUL!
2,00	3,00	2,00	2,00	2,00	2,00	#NUL!	#NUL!	#NUL!
2,00	3,00	2,00	2,00	2,00	2,00	#NUL!	#NUL!	#NUL!
2,00	1,00	2,00	2,00	2,00	2,00	#NUL!	#NUL!	#NUL!
2,00	3,00	2,00	2,00	2,00	2,00	#NUL!	#NUL!	#NUL!
2,00	3,00	2,00	2,00	2,00	2,00	#NUL!	#NUL!	#NUL!
2,00	1,00	1,00	2,00	2,00	2,00	#NUL!	#NUL!	#NUL!

année_dm	ATCD_CM(	NYHA	valv_avant	valv_aucoı	termedecc	decouverte	sinusal	ACFA
#NUL!	2,00	3,00	1,00	2,00	#NUL!	#NUL!	2,00	1,00
#NUL!	2,00	2,00	1,00	2,00	#NUL!	#NUL!	1,00	2,00
#NUL!	2,00	1,00	2,00	1,00	27,00	#NUL!	1,00	2,00
#NUL!	2,00	1,00	1,00	2,00	#NUL!	#NUL!	2,00	1,00
#NUL!	2,00	1,00	2,00	1,00	16,00	#NUL!	2,00	2,00
#NUL!	2,00	2,00	2,00	1,00	25,00	#NUL!	2,00	1,00
#NUL!	2,00	2,00	1,00	2,00	#NUL!	#NUL!	1,00	2,00
#NUL!	2,00	1,00	1,00	2,00	#NUL!	1,00	1,00	2,00
#NUL!	2,00	1,00	1,00	2,00	#NUL!	#NUL!	1,00	2,00
#NUL!	2,00	2,00	1,00	2,00	#NUL!	#NUL!	1,00	2,00
#NUL!	2,00	1,00	1,00	2,00	#NUL!	#NUL!	2,00	1,00
#NUL!	2,00	1,00	1,00	2,00	#NUL!	#NUL!	2,00	1,00
#NUL!	2,00	1,00	2,00	1,00	26,00	#NUL!	1,00	1,00
#NUL!	2,00	1,00	1,00	2,00	#NUL!	#NUL!	1,00	2,00
#NUL!	2,00	2,00	2,00	1,00	16,00	#NUL!	1,00	2,00
#NUL!	2,00	2,00	1,00	2,00	#NUL!	#NUL!	1,00	2,00
#NUL!	2,00	1,00	1,00	2,00	#NUL!	#NUL!	1,00	2,00
#NUL!	2,00	1,00	1,00	2,00	#NUL!	#NUL!	1,00	2,00
#NUL!	#NUL!	1,00	2,00	1,00	26,00	2,00	2,00	1,00
#NUL!	2,00	2,00	1,00	2,00	32,00	#NUL!	1,00	2,00
#NUL!	2,00	1,00	1,00	2,00	#NUL!	#NUL!	1,00	2,00
#NUL!	2,00	2,00	2,00	1,00	32,00	#NUL!	2,00	1,00
#NUL!	2,00	1,00	1,00	2,00	#NUL!	#NUL!	2,00	1,00
#NUL!	2,00	1,00	2,00	1,00	30,00	#NUL!	1,00	2,00
#NUL!	2,00	2,00	1,00	2,00	#NUL!	#NUL!	1,00	2,00
#NUL!	2,00	1,00	2,00	1,00	30,00	1,00	1,00	2,00
#NUL!	2,00	1,00	2,00	1,00	36,00	#NUL!	1,00	2,00
#NUL!	2,00	2,00	2,00	1,00	#NUL!	#NUL!	1,00	2,00
#NUL!	2,00	1,00	2,00	1,00	28,00	#NUL!	1,00	2,00
#NUL!	2,00	2,00	2,00	1,00	24,00	#NUL!	2,00	1,00
#NUL!	2,00	2,00	2,00	1,00	26,00	#NUL!	1,00	2,00
#NUL!	2,00	2,00	1,00	2,00	#NUL!	#NUL!	2,00	1,00
#NUL!	2,00	3,00	2,00	1,00	36,00	#NUL!	1,00	2,00
#NUL!	2,00	2,00	1,00	2,00	#NUL!	#NUL!	1,00	2,00
#NUL!	2,00	2,00	2,00	1,00	28,00	#NUL!	1,00	2,00
#NUL!	2,00	2,00	1,00	2,00	#NUL!	#NUL!	2,00	1,00
#NUL!	2,00	2,00	2,00	1,00	33,00	2,00	2,00	1,00
#NUL!	2,00	1,00	1,00	2,00	#NUL!	#NUL!	1,00	2,00
2003,00	2,00	2,00	1,00	2,00	#NUL!	#NUL!	2,00	1,00
#NUL!	2,00	2,00	1,00	2,00	#NUL!	#NUL!	2,00	1,00
#NUL!	2,00	2,00	1,00	2,00	#NUL!	#NUL!	2,00	1,00
#NUL!	2,00	1,00	2,00	1,00	32,00	#NUL!	1,00	2,00
#NUL!	2,00	1,00	2,00	1,00	38,00	#NUL!	1,00	2,00
#NUL!	2,00	1,00	1,00	2,00	#NUL!	#NUL!	1,00	2,00
#NUL!	2,00	3,00	2,00	1,00	35,00	#NUL!	2,00	1,00
#NUL!	2,00	2,00	2,00	1,00	20,00	#NUL!	1,00	2,00
#NUL!	2,00	3,00	1,00	2,00	#NUL!	#NUL!	2,00	1,00
#NUL!	2,00	1,00	2,00	1,00	36,00	#NUL!	1,00	2,00
#NUL!	2,00	3,00	2,00	1,00	16,00	#NUL!	1,00	2,00

#NUL!	2,00	2,00	1,00	2,00	#NUL!	#NUL!	2,00	1,00
#NUL!	2,00	2,00	2,00	1,00	28,00	#NUL!	2,00	1,00
#NUL!	2,00	1,00	2,00	1,00	8,00	#NUL!	1,00	2,00
#NUL!	2,00	1,00	1,00	2,00	#NUL!	#NUL!	1,00	2,00
#NUL!	2,00	1,00	2,00	1,00	24,00	1,00	1,00	2,00
#NUL!	2,00	1,00	1,00	2,00	#NUL!	#NUL!	2,00	1,00
#NUL!	2,00	2,00	2,00	1,00	28,00	#NUL!	2,00	1,00
#NUL!	2,00	1,00	2,00	1,00	32,00	#NUL!	1,00	2,00
#NUL!	2,00	2,00	2,00	1,00	12,00	#NUL!	1,00	2,00
#NUL!	2,00	2,00	2,00	1,00	35,00	#NUL!	2,00	1,00
#NUL!	2,00	1,00	2,00	1,00	28,00	#NUL!	1,00	2,00

Anemie	HB	termeETT	FeVG	DTDVG	DTSVG	PAPS	DiamètreC	polyvalvulc
1,00	10,1	39,00	60,00	44,00	29,00	70,00	62,00	2,00
2,00	11,0	13,00	60,00	50,00	33,00	36,00	61,00	2,00
2,00	11,0	27,00	60,00	41,00	28,00	55,00	43,00	1,00
1,00	9,0	35,00	50,00	50,00	31,00	35,00	57,00	2,00
1,00	9,7	27,00	65,00	45,00	30,00	60,00	58,00	2,00
2,00	11,1	25,00	50,00	55,00	36,00	55,00	50,00	1,00
1,00	12,2	32,00	60,00	56,00	36,00	35,00	49,00	2,00
2,00	11,7	38,00	60,00	59,00	40,00	30,00	42,00	1,00
2,00	12,0	35,00	60,00	45,00	25,00	45,00	44,00	2,00
1,00	9,6	36,00	60,00	48,00	32,00	60,00	54,00	1,00
1,00	10,0	38,00	60,00	40,00	25,00	70,00	58,00	1,00
1,00	11,0	26,00	60,00	48,00	25,00	50,00	50,00	1,00
1,00	11,3	26,00	60,00	48,00	21,00	38,00	39,00	2,00
1,00	10,0	22,00	65,00	52,00	35,00	35,00	52,00	2,00
1,00	8,7	30,00	60,00	57,00	36,00	45,00	47,00	2,00
2,00	12,0	17,00	66,00	53,00	33,00	42,00	47,00	2,00
2,00	11,0	32,00	60,00	50,00	35,00	20,00	40,00	2,00
1,00	9,3	33,00	60,00	48,00	29,00	28,00	51,00	1,00
2,00	11,0	26,00	60,00	45,00	29,00	42,00	57,00	2,00
1,00	9,0	32,00	60,00	45,00	25,00	41,00	46,00	1,00
1,00	9,0	32,00	60,00	45,00	25,00	35,00	45,00	2,00
2,00	11,0	32,00	60,00	35,00	24,00	70,00	65,00	2,00
2,00	12,0	39,00	60,00	51,00	39,00	51,00	51,00	1,00
1,00	9,0	30,00	60,00	48,00	28,00	29,00	45,00	2,00
1,00	9,5	33,00	60,00	49,00	30,00	45,00	49,00	1,00
1,00	7,0	#NUL!	50,00	60,00	45,00	70,00	52,00	1,00
1,00	9,0	36,00	60,00	52,00	31,00	50,00	46,00	1,00
2,00	12,0	#NUL!	25,00	65,00	53,00	85,00	64,00	1,00
2,00	11,3	28,00	60,00	45,00	25,00	38,00	58,00	1,00
1,00	10,0	25,00	60,00	46,00	26,00	63,00	53,00	2,00
1,00	9,2	26,00	50,00	41,00	26,00	70,00	45,00	2,00
1,00	9,0	27,00	60,00	46,00	29,00	45,00	51,00	1,00
1,00	9,0	36,00	60,00	55,00	26,00	40,00	40,00	2,00
2,00	11,9	24,00	60,00	57,00	40,00	35,00	52,00	2,00
1,00	10,0	29,00	60,00	55,00	33,00	55,00	60,00	2,00
1,00	10,0	32,00	60,00	49,00	37,00	75,00	58,00	2,00
2,00	12,4	33,00	55,00	45,00	33,00	55,00	58,00	1,00
2,00	11,5	30,00	65,00	49,00	34,00	65,00	48,00	1,00
1,00	9,0	29,00	65,00	50,00	35,00	55,00	55,00	1,00
1,00	9,4	12,00	65,00	56,00	42,00	63,00	53,00	1,00
1,00	9,7	34,00	65,00	52,00	35,00	42,00	52,00	1,00
2,00	11,0	#NUL!	60,00	49,00	31,00	69,00	52,00	1,00
1,00	9,2	38,00	45,00	59,00	42,00	57,00	48,00	2,00
1,00	10,4	34,00	60,00	52,00	35,00	50,00	50,00	1,00
2,00	11,5	35,00	60,00	42,00	22,00	95,00	60,00	2,00
2,00	12,0	24,00	60,00	48,00	34,00	38,00	45,00	1,00
1,00	9,3	37,00	60,00	47,00	34,00	62,00	54,00	2,00
2,00	11,0	39,00	60,00	55,00	35,00	30,00	37,00	2,00
1,00	10,0	17,00	60,00	33,00	18,00	55,00	48,00	2,00

2,00	12,7	33,00	55,00	45,00	25,00	75,00	52,00	2,00
2,00	11,0	28,00	60,00	45,00	25,00	65,00	53,00	1,00
1,00	9,0	9,00	55,00	46,00	31,00	50,00	32,00	1,00
2,00	11,0	28,00	65,00	45,00	25,00	39,00	42,00	2,00
1,00	9,2	24,00	50,00	48,00	33,00	65,00	54,00	1,00
1,00	9,4	33,00	60,00	50,00	35,00	30,00	45,00	1,00
2,00	12,3	28,00	60,00	66,00	44,00	40,00	55,00	2,00
1,00	10,0	33,00	60,00	51,00	33,00	50,00	47,00	2,00
1,00	9,0	19,00	65,00	45,00	30,00	36,00	44,00	2,00
1,00	9,0	35,00	60,00	69,00	47,00	80,00	71,00	2,00
1,00	9,0	28,00	60,00	45,00	26,00	35,00	45,00	1,00

RM	RM_enton	severite_R	surfacemit	Gradient	IM	severiteIM	mecanisme	SOR_IM
1,00	#NUL!	1,00	0,90	16,00	2,00	#NUL!		#NUL!
1,00	2,00	1,00	1,38	6,00	1,00	1,00	restriction	53,00
1,00	#NUL!	1,00	1,20	25,00	2,00	#NUL!		#NUL!
1,00	2,00	1,00	1,30	16,00	2,00	#NUL!		#NUL!
1,00	2,00	1,00	1,20	20,00	2,00	3,00	restriction	5,00
1,00	#NUL!	1,00	1,20	20,00	2,00	#NUL!		#NUL!
2,00	#NUL!	#NUL!	#NUL!	#NUL!	1,00	1,00	restriction	38,00
2,00	#NUL!	#NUL!	#NUL!	#NUL!	1,00	3,00	ischemique	13,00
1,00	2,00	1,00	1,40	14,00	2,00	#NUL!		#NUL!
1,00	#NUL!	1,00	1,70	20,00	1,00	3,00	restriction	12,00
1,00	2,00	1,00	1,00	15,00	1,00	2,00	restriction	25,00
1,00	1,00	1,00	1,40	15,00	1,00	3,00	restriction	25,00
2,00	#NUL!	#NUL!	#NUL!	#NUL!	2,00	#NUL!		#NUL!
1,00	#NUL!	1,00	1,30	11,00	1,00	3,00	rstriction d	15,00
1,00	#NUL!	1,00	1,20	18,00	1,00	3,00	restriction	12,00
2,00	#NUL!	#NUL!	#NUL!	#NUL!	1,00	1,00	restriction	40,00
2,00	#NUL!	#NUL!	#NUL!	#NUL!	2,00	#NUL!		#NUL!
1,00	2,00	1,00	1,00	1,20	2,00	#NUL!		#NUL!
1,00	1,00	1,00	1,30	15,00	2,00	#NUL!		#NUL!
1,00	1,00	1,00	1,70	24,00	1,00	4,00		#NUL!
1,00	2,00	1,00	1,40	11,00	2,00	#NUL!		#NUL!
1,00	#NUL!	1,00	0,90	15,00	2,00	#NUL!		#NUL!
1,00	1,00	1,00	1,70	16,00	2,00	#NUL!		#NUL!
1,00	2,00	1,00	1,10	15,00	2,00	#NUL!		#NUL!
1,00	1,00	1,00	1,60	16,00	1,00	3,00	restriction	11,00
1,00	2,00	3,00	2,00	8,00	1,00	1,00	prolapsus	42,00
1,00	1,00	1,00	1,60	19,00	1,00	2,00	restriction	28,00
1,00	2,00	1,00	0,70	26,00	1,00	3,00	restrictionl	12,00
1,00	2,00	1,00	1,30	16,00	2,00	#NUL!		#NUL!
1,00	2,00	1,00	1,10	21,00	2,00	#NUL!		#NUL!
1,00	2,00	1,00	1,00	30,00	2,00	#NUL!		#NUL!
1,00	2,00	1,00	1,20	10,00	1,00	3,00	restriction	22,00
2,00	#NUL!	#NUL!	#NUL!	#NUL!	2,00	#NUL!		#NUL!
2,00	#NUL!	#NUL!	#NUL!	#NUL!	1,00	1,00	restriction	40,00
1,00	1,00	1,00	1,00	21,00	2,00	#NUL!		#NUL!
1,00	2,00	1,00	1,00	14,00	1,00	3,00	restriction	21,00
1,00	2,00	1,00	1,20	18,00	1,00	3,00	restriction	11,00
1,00	2,00	1,00	1,50	17,00	1,00	2,00	restriction	31,00
1,00	2,00	2,00	1,70	9,00	1,00	1,00	restriction	42,00
1,00	2,00	1,00	1,00	13,00	1,00	3,00	restriction	13,00
1,00	2,00	1,00	1,50	13,00	1,00	2,00	retstrictior	24,00
1,00	2,00	1,00	1,20	14,00	1,00	3,00	restriction	13,00
1,00	2,00	1,00	1,30	21,00	1,00	1,00	restriction	42,00
1,00	1,00	1,00	1,60	15,00	1,00	4,00	restrictioor	#NUL!
1,00	2,00	1,00	1,00	22,00	2,00	#NUL!		#NUL!
1,00	2,00	1,00	1,50	10,00	1,00	3,00	restriction	11,00
1,00	2,00	1,00	1,00	#NUL!	2,00	#NUL!		#NUL!
2,00	#NUL!	#NUL!	#NUL!	#NUL!	2,00	#NUL!		#NUL!
1,00	2,00	1,00	0,90	28,00	2,00	#NUL!		#NUL!

1,00	2,00	1,00	1,00	16,00	2,00	#NUL!		#NUL!
1,00	1,00	1,00	1,00	24,00	1,00	3,00	restriction	12,00
2,00	#NUL!	#NUL!	#NUL!	#NUL!	2,00	3,00	restriction	11,00
2,00	#NUL!	#NUL!	#NUL!	#NUL!	1,00	1,00	restriction	42,00
1,00	2,00	1,00	1,00	20,00	1,00	3,00	restriction	11,00
2,00	#NUL!	#NUL!	#NUL!	#NUL!	2,00	#NUL!		#NUL!
2,00	#NUL!	#NUL!	#NUL!	#NUL!	1,00	1,00	restriction	71,00
1,00	#NUL!	1,00	1,20	20,00	2,00	#NUL!		#NUL!
1,00	2,00	1,00	1,20	12,00	2,00	#NUL!		#NUL!
2,00	#NUL!	#NUL!	#NUL!	#NUL!	1,00	1,00	prolapsus /	127,00
2,00	#NUL!	#NUL!	#NUL!	#NUL!	2,00	#NUL!		#NUL!

VR_IM	RAo	severite_R	surfaceaor	GradientA	IA	severite_I	SOR_IA	VR_IA
#NUL!	2,00	#NUL!	#NUL!	#NUL!	2,00	#NUL!	#NUL!	#NUL!
65,00	1,00	2,00	1,37	23,00	1,00	3,00	21,00	25,00
#NUL!	2,00	#NUL!	#NUL!	#NUL!	2,00	#NUL!	#NUL!	#NUL!
#NUL!	2,00	#NUL!	#NUL!	#NUL!	2,00	#NUL!	#NUL!	#NUL!
#NUL!	2,00	#NUL!	#NUL!	#NUL!	2,00	#NUL!	#NUL!	#NUL!
#NUL!	1,00	1,00	#NUL!	53,00	1,00	2,00	#NUL!	#NUL!
60,00	2,00	#NUL!	#NUL!	#NUL!	2,00	#NUL!	#NUL!	#NUL!
31,00	1,00	1,00	0,60	84,00	1,00	2,00	19,00	45,00
#NUL!	2,00	#NUL!	#NUL!	#NUL!	2,00	#NUL!	#NUL!	#NUL!
26,00	2,00	#NUL!	#NUL!	#NUL!	2,00	#NUL!	#NUL!	#NUL!
40,00	2,00	#NUL!	#NUL!	#NUL!	2,00	#NUL!	#NUL!	#NUL!
36,00	2,00	#NUL!	#NUL!	#NUL!	2,00	#NUL!	#NUL!	#NUL!
#NUL!	1,00	1,00	#NUL!	#NUL!	1,00	2,00	26,00	54,00
26,00	1,00	1,00	#NUL!	55,00	2,00	3,00	9,00	18,00
23,00	1,00	2,00	1,17	39,00	1,00	1,00	30,00	60,00
60,00	2,00	#NUL!	#NUL!	#NUL!	2,00	#NUL!	#NUL!	#NUL!
#NUL!	2,00	#NUL!	#NUL!	#NUL!	2,00	#NUL!	#NUL!	#NUL!
#NUL!	2,00	#NUL!	#NUL!	#NUL!	2,00	#NUL!	#NUL!	#NUL!
#NUL!	2,00	#NUL!	#NUL!	#NUL!	2,00	#NUL!	#NUL!	#NUL!
#NUL!	1,00	3,00	1,50	25,00	2,00	#NUL!	#NUL!	#NUL!
#NUL!	2,00	#NUL!	#NUL!	#NUL!	2,00	#NUL!	#NUL!	#NUL!
#NUL!	2,00	#NUL!	#NUL!	#NUL!	2,00	#NUL!	#NUL!	#NUL!
#NUL!	2,00	#NUL!	#NUL!	#NUL!	2,00	#NUL!	#NUL!	#NUL!
#NUL!	2,00	#NUL!	#NUL!	#NUL!	2,00	#NUL!	#NUL!	#NUL!
25,00	2,00	#NUL!	#NUL!	#NUL!	2,00	#NUL!	#NUL!	#NUL!
65,00	2,00	#NUL!	#NUL!	#NUL!	2,00	#NUL!	#NUL!	#NUL!
45,00	2,00	#NUL!	#NUL!	#NUL!	2,00	#NUL!	#NUL!	#NUL!
24,00	2,00	#NUL!	#NUL!	#NUL!	1,00	3,00	28,00	36,00
#NUL!	2,00	#NUL!	#NUL!	#NUL!	2,00	#NUL!	#NUL!	#NUL!
#NUL!	2,00	#NUL!	#NUL!	#NUL!	2,00	#NUL!	#NUL!	#NUL!
#NUL!	2,00	#NUL!	#NUL!	#NUL!	2,00	#NUL!	#NUL!	#NUL!
32,00	1,00	3,00	1,85	23,00	1,00	2,00	22,00	35,00
#NUL!	1,00	1,00	#NUL!	114,00	2,00	#NUL!	#NUL!	#NUL!
60,00	2,00	#NUL!	#NUL!	#NUL!	2,00	#NUL!	#NUL!	#NUL!
#NUL!	2,00	#NUL!	#NUL!	#NUL!	2,00	#NUL!	#NUL!	#NUL!
29,00	2,00	#NUL!	#NUL!	#NUL!	2,00	#NUL!	#NUL!	#NUL!
15,00	2,00	#NUL!	#NUL!	#NUL!	2,00	#NUL!	#NUL!	#NUL!
45,00	1,00	2,00	1,10	47,00	1,00	3,00	18,00	35,00
69,00	1,00	1,00	1,00	55,00	1,00	4,00	#NUL!	#NUL!
28,00	1,00	1,00	0,90	50,00	1,00	3,00	12,00	23,00
40,00	2,00	#NUL!	#NUL!	#NUL!	2,00	#NUL!	#NUL!	#NUL!
20,00	2,00	#NUL!	#NUL!	#NUL!	2,00	#NUL!	#NUL!	#NUL!
64,00	2,00	#NUL!	#NUL!	#NUL!	2,00	#NUL!	#NUL!	#NUL!
#NUL!	2,00	#NUL!	#NUL!	#NUL!	1,00	3,00	15,00	25,00
#NUL!	2,00	#NUL!	#NUL!	#NUL!	2,00	#NUL!	#NUL!	#NUL!
14,00	2,00	#NUL!	#NUL!	#NUL!	1,00	3,00	13,00	28,00
#NUL!	2,00	#NUL!	#NUL!	#NUL!	2,00	#NUL!	#NUL!	#NUL!
#NUL!	1,00	1,00	1,00	55,00	2,00	#NUL!	#NUL!	#NUL!
#NUL!	2,00	#NUL!	#NUL!	#NUL!	2,00	#NUL!	#NUL!	#NUL!

#NUL!	2,00	#NUL!	#NUL!	#NUL!	2,00	#NUL!	#NUL!	#NUL!
25,00	2,00	#NUL!	#NUL!	#NUL!	2,00	#NUL!	#NUL!	#NUL!
18,00	1,00	1,00	0,60	55,00	1,00	1,00	37,00	65,00
65,00	2,00	#NUL!	#NUL!	#NUL!	2,00	#NUL!	#NUL!	#NUL!
12,00	2,00	#NUL!	#NUL!	#NUL!	1,00	23,00	29,00	#NUL!
#NUL!	1,00	1,00	#NUL!	64,00	2,00	#NUL!	#NUL!	#NUL!
104,00	2,00	#NUL!	#NUL!	#NUL!	2,00	#NUL!	#NUL!	#NUL!
#NUL!	2,00	#NUL!	#NUL!	#NUL!	2,00	#NUL!	#NUL!	#NUL!
#NUL!	2,00	#NUL!	#NUL!	#NUL!	2,00	#NUL!	#NUL!	#NUL!
139,00	2,00	#NUL!	#NUL!	#NUL!	2,00	#NUL!	#NUL!	#NUL!
#NUL!	1,00	1,00	#NUL!	74,00	2,00	#NUL!	#NUL!	#NUL!

IT	severite_IT	Avk	aspegic	lasilix	IEC	ARRET_IEC	BETABLOQ	ARRET_BB
1,00	1,00	#NUL!	#NUL!	#NUL!	#NUL!	#NUL!	#NUL!	#NUL!
2,00	2,00	1,00	2,00	1,00	2,00	#NUL!	2,00	#NUL!
1,00	3,00	2,00	2,00	1,00	2,00	#NUL!	2,00	#NUL!
1,00	4,00	1,00	2,00	1,00	2,00	#NUL!	2,00	#NUL!
1,00	3,00	2,00	2,00	2,00	2,00	#NUL!	2,00	#NUL!
1,00	2,00	2,00	2,00	1,00	2,00	#NUL!	2,00	#NUL!
1,00	4,00	2,00	2,00	2,00	2,00	#NUL!	2,00	#NUL!
1,00	4,00	2,00	1,00	2,00	1,00	20,00	1,00	2,00
1,00	3,00	2,00	2,00	2,00	2,00	#NUL!	2,00	#NUL!
1,00	3,00	2,00	2,00	1,00	2,00	#NUL!	1,00	2,00
1,00	2,00	1,00	2,00	1,00	2,00	#NUL!	1,00	1,00
1,00	33,00	1,00	2,00	1,00	2,00	#NUL!	1,00	#NUL!
1,00	4,00	2,00	2,00	2,00	2,00	#NUL!	1,00	#NUL!
1,00	4,00	2,00	2,00	#NUL!	2,00	#NUL!	2,00	#NUL!
1,00	3,00	2,00	2,00	2,00	2,00	#NUL!	2,00	#NUL!
2,00	#NUL!	2,00	#NUL!	2,00	2,00	#NUL!	2,00	#NUL!
2,00	#NUL!	2,00	2,00	2,00	2,00	#NUL!	2,00	#NUL!
1,00	3,00	2,00	2,00	1,00	2,00	#NUL!	2,00	#NUL!
1,00	3,00	1,00	2,00	1,00	2,00	#NUL!	1,00	#NUL!
1,00	2,00	2,00	2,00	1,00	2,00	#NUL!	2,00	#NUL!
1,00	3,00	2,00	2,00	2,00	2,00	#NUL!	2,00	#NUL!
1,00	3,00	1,00	1,00	2,00	2,00	#NUL!	1,00	2,00
1,00	4,00	1,00	2,00	2,00	2,00	#NUL!	2,00	#NUL!
1,00	4,00	2,00	2,00	1,00	2,00	#NUL!	2,00	#NUL!
1,00	3,00	2,00	2,00	1,00	2,00	#NUL!	2,00	#NUL!
1,00	2,00	2,00	2,00	2,00	2,00	#NUL!	2,00	#NUL!
1,00	3,00	2,00	2,00	2,00	2,00	#NUL!	2,00	#NUL!
1,00	2,00	2,00	2,00	2,00	2,00	#NUL!	2,00	#NUL!
1,00	3,00	2,00	2,00	2,00	2,00	#NUL!	2,00	#NUL!
1,00	3,00	1,00	2,00	1,00	1,00	25,00	2,00	#NUL!
1,00	3,00	2,00	2,00	1,00	2,00	#NUL!	2,00	#NUL!
1,00	3,00	1,00	2,00	1,00	1,00	1,00	1,00	1,00
1,00	4,00	2,00	2,00	1,00	2,00	#NUL!	1,00	#NUL!
1,00	4,00	2,00	2,00	2,00	2,00	#NUL!	2,00	#NUL!
1,00	3,00	2,00	2,00	2,00	2,00	#NUL!	2,00	#NUL!
1,00	3,00	1,00	2,00	1,00	2,00	#NUL!	2,00	#NUL!
1,00	2,00	2,00	2,00	1,00	2,00	#NUL!	1,00	#NUL!
1,00	2,00	2,00	2,00	1,00	2,00	#NUL!	2,00	#NUL!
1,00	3,00	1,00	2,00	1,00	2,00	#NUL!	2,00	#NUL!
1,00	3,00	1,00	2,00	1,00	2,00	#NUL!	1,00	1,00
1,00	3,00	1,00	2,00	2,00	2,00	#NUL!	2,00	#NUL!
1,00	2,00	2,00	2,00	1,00	2,00	#NUL!	1,00	2,00
1,00	3,00	2,00	2,00	1,00	2,00	#NUL!	2,00	#NUL!
1,00	4,00	2,00	2,00	2,00	2,00	#NUL!	2,00	#NUL!
1,00	1,00	2,00	2,00	1,00	2,00	#NUL!	2,00	#NUL!
1,00	4,00	2,00	2,00	2,00	2,00	#NUL!	2,00	#NUL!
1,00	2,00	1,00	2,00	2,00	2,00	#NUL!	2,00	#NUL!
1,00	1,00	2,00	2,00	2,00	2,00	#NUL!	2,00	#NUL!
1,00	3,00	2,00	2,00	1,00	2,00	#NUL!	2,00	#NUL!

1,00	1,00	1,00	2,00	1,00	2,00	#NUL!	2,00	#NUL!
1,00	3,00	2,00	2,00	2,00	2,00	#NUL!	2,00	#NUL!
1,00	4,00	2,00	2,00	1,00	2,00	#NUL!	2,00	#NUL!
1,00	3,00	2,00	2,00	1,00	1,00	1,00	2,00	#NUL!
1,00	3,00	2,00	2,00	2,00	2,00	#NUL!	2,00	#NUL!
1,00	3,00	1,00	2,00	2,00	2,00	#NUL!	1,00	2,00
1,00	4,00	1,00	2,00	#NUL!	#NUL!	#NUL!	2,00	#NUL!
1,00	4,00	2,00	2,00	1,00	2,00	#NUL!	2,00	#NUL!
1,00	3,00	2,00	#NUL!	1,00	2,00	#NUL!	2,00	#NUL!
1,00	4,00	1,00	2,00	1,00	2,00	#NUL!	2,00	#NUL!
1,00	4,00	2,00	2,00	2,00	2,00	#NUL!	2,00	#NUL!

GROSSESSI	TERME_GY	TERME_CA	GROSSESSI	TERMEACC	VOIEBASSE	BASSE_DE(	BASSE_FOI	CESARENN
#NUL!	#NUL!	#NUL!	#NUL!	38,00	2,00	2,00	2,00	1,00
1,00	10,00	10,00	2,00	37,00	2,00	2,00	2,00	1,00
1,00	9,00	27,00	2,00	39,00	1,00	2,00	2,00	#NUL!
1,00	#NUL!	#NUL!	2,00	38,00	2,00	2,00	2,00	1,00
1,00	6,00	16,00	1,00	30,00	2,00	2,00	2,00	1,00
1,00	#NUL!	25,00	2,00	38,00	2,00	2,00	2,00	2,00
1,00	8,00	28,00	2,00	38,00	1,00	2,00	2,00	#NUL!
1,00	8,00	18,00	2,00	37,00	2,00	2,00	2,00	1,00
2,00	#NUL!	#NUL!	2,00	39,00	2,00	2,00	2,00	1,00
1,00	9,00	10,00	2,00	41,00	1,00	2,00	2,00	2,00
1,00	8,00	8,00	2,00	39,00	2,00	2,00	2,00	2,00
1,00	8,00	26,00	2,00	35,00	2,00	2,00	2,00	1,00
1,00	8,00	26,00	2,00	38,00	1,00	2,00	2,00	#NUL!
#NUL!	#NUL!	#NUL!	#NUL!	#NUL!	2,00	2,00	2,00	1,00
1,00	#NUL!	#NUL!	2,00	38,00	1,00	2,00	2,00	#NUL!
1,00	#NUL!	3,00	2,00	38,00	2,00	2,00	2,00	1,00
1,00	8,00	19,00	2,00	38,00	2,00	2,00	2,00	1,00
1,00	10,00	32,00	2,00	38,00	1,00	2,00	2,00	#NUL!
1,00	8,00	26,00	2,00	37,00	1,00	2,00	2,00	#NUL!
1,00	8,00	9,00	2,00	33,00	2,00	2,00	2,00	1,00
1,00	#NUL!	#NUL!	2,00	38,00	2,00	2,00	2,00	1,00
1,00	#NUL!	32,00	2,00	39,00	2,00	2,00	2,00	1,00
1,00	#NUL!	#NUL!	2,00	39,00	2,00	2,00	2,00	1,00
2,00	12,00	30,00	2,00	38,00	1,00	2,00	2,00	#NUL!
1,00	8,00	12,00	2,00	38,00	2,00	2,00	2,00	1,00
1,00	6,00	#NUL!	2,00	38,00	1,00	2,00	2,00	#NUL!
1,00	9,00	38,00	2,00	37,00	2,00	2,00	2,00	1,00
1,00	#NUL!	#NUL!	2,00	39,00	1,00	2,00	2,00	3,00
1,00	8,00	28,00	2,00	38,00	2,00	1,00	2,00	#NUL!
2,00	#NUL!	#NUL!	2,00	38,00	1,00	2,00	2,00	#NUL!
2,00	26,00	26,00	2,00	38,00	1,00	2,00	2,00	#NUL!
2,00	25,00	29,00	2,00	35,00	2,00	2,00	2,00	1,00
2,00	#NUL!	#NUL!	2,00	36,00	2,00	2,00	2,00	1,00
1,00	#NUL!	#NUL!	2,00	39,00	1,00	2,00	2,00	2,00
2,00	#NUL!	#NUL!	2,00	38,00	1,00	2,00	2,00	#NUL!
1,00	12,00	25,00	2,00	39,00	2,00	2,00	2,00	1,00
1,00	8,00	33,00	2,00	34,00	2,00	2,00	2,00	1,00
1,00	10,00	22,00	2,00	35,00	2,00	2,00	2,00	1,00
2,00	#NUL!	#NUL!	2,00	38,00	2,00	2,00	2,00	1,00
1,00	8,00	12,00	2,00	38,00	2,00	2,00	2,00	1,00
1,00	8,00	15,00	2,00	37,00	2,00	2,00	2,00	1,00
1,00	10,00	30,00	2,00	39,00	1,00	2,00	2,00	3,00
1,00	2,00	9,00	2,00	38,00	2,00	2,00	2,00	1,00
1,00	#NUL!	#NUL!	2,00	38,00	2,00	2,00	2,00	1,00
1,00	#NUL!	#NUL!	2,00	35,00	2,00	2,00	2,00	1,00
1,00	12,00	20,00	2,00	38,00	2,00	2,00	2,00	1,00
1,00	#NUL!	#NUL!	2,00	40,00	2,00	2,00	2,00	1,00
1,00	#NUL!	#NUL!	2,00	39,00	2,00	2,00	2,00	1,00
1,00	12,00	16,00	2,00	37,00	1,00	2,00	2,00	#NUL!

1,00	#NUL!	#NUL!	2,00	35,00	2,00	2,00	2,00	1,00
1,00	8,00	28,00	2,00	35,00	2,00	2,00	2,00	1,00
1,00	9,00	9,00	2,00	35,00	2,00	2,00	2,00	1,00
1,00	9,00	29,00	2,00	39,00	1,00	2,00	2,00	#NUL!
1,00	6,00	#NUL!	2,00	2,00	2,00	2,00	2,00	1,00
2,00	#NUL!	#NUL!	2,00	39,00	2,00	2,00	2,00	1,00
#NUL!	#NUL!	#NUL!	#NUL!	#NUL!	#NUL!	#NUL!	#NUL!	#NUL!
1,00	9,00	33,00	2,00	37,00	2,00	2,00	2,00	1,00
1,00	10,00	12,00	2,00	39,00	1,00	2,00	2,00	2,00
2,00	#NUL!	#NUL!	#NUL!	35,00	2,00	2,00	2,00	1,00
1,00	9,00	28,00	2,00	37,00	2,00	2,00	2,00	1,00

INDICATIO	anesthesie	DELIVRAN(	DUREE_HC	SEXE_NN	POIDS_NA	APGAR	comemnta	CPC_MATE
	#NUL!	#NUL!	#NUL!	#NUL!	#NUL!	#NUL!		#NUL!
htap cardia	2,00	33,00	5,00	2,00	3000	8,00		1,00
	#NUL!	#NUL!	3,00	2,00	3400	9,00		1,00
thrombope	1,00	3,00	18,00	1,00	3400	9,00		1,00
premat HT	1,00	3,00	16,00	2,00	1500	8,00		2,00
UC+valvulo	2,00	2,00	7,00	2,00	2950	9,00		1,00
	2,00	2,00	3,00	2,00	3000	9,00		2,00
rao serré c	2,00	3,00	6,00	2,00	3200	9,00		2,00
HTAP RM S	2,00	3,00	3,00	1,00	3000	9,00		2,00
	2,00	1,00	2,00	2,00	3200	9,00		1,00
uterus cica	#NUL!	#NUL!	#NUL!	#NUL!	#NUL!	#NUL!		#NUL!
rm serré ht	1,00	1,00	7,00	2,00	3100	8,00		1,00
	#NUL!	#NUL!	3,00	1,00	3070	9,00		2,00
RAao serré	2,00	3,00	3,00	2,00	3200	9,00		1,00
	#NUL!	#NUL!	3,00	2,00	3100	9,00		2,00
uterux bici	1,00	3,00	3,00	1,00	3750	5,00	cyanose, b	2,00
RP tres ser	2,00	3,00	3,00	2,00	3200	9,00		2,00
	#NUL!	2,00	2,00	2,00	3300	9,00		1,00
	#NUL!	#NUL!	8,00	2,00	2400	8,00		1,00
travail+dec	2,00	3,00	4,00	1,00	2000	8,00		1,00
uterus bici	1,00	3,00	3,00	2,00	3100	9,00		2,00
RM+HTAP	1,00	3,00	15,00	2,00	2500	9,00		1,00
RM+HTAP	2,00	3,00	7,00	1,00	3100	9,00		2,00
	2,00	2,00	3,00	2,00	3000	9,00		2,00
htap rm se	2,00	3,00	3,00	2,00	3200	9,00		2,00
	#NUL!	#NUL!	#NUL!	2,00	3000	9,00		1,00
htap	2,00	2,00	4,00	2,00	2400	8,00	puis10/10	2,00
	#NUL!	#NUL!	14,00	2,00	2200	9,00		1,00
	#NUL!	2,00	3,00	2,00	3100	9,00		1,00
	#NUL!	#NUL!	6,00	1,00	3000	9,00		1,00
	#NUL!	#NUL!	3,00	2,00	2750	9,00		1,00
cicatricel+l	2,00	3,00	8,00	2,00	2900	8,00		1,00
RA tres ser	1,00	3,00	4,00	1,00	3000	9,00		1,00
	#NUL!	#NUL!	#NUL!	1,00	3000	9,00		2,00
	#NUL!	#NUL!	3,00	2,00	3000	9,00		2,00
rm htap	2,00	3,00	8,00	1,00	2500	8,00		1,00
RPM souffr	2,00	3,00	10,00	2,00	2300	5,00		1,00
RM serré H	2,00	3,00	5,00	2,00	2400	8,00		2,00
htap	22,00	2,00	8,00	2,00	2500	8,00		1,00
rm serré ra	2,00	3,00	6,00	2,00	3200	8,00		1,00
rm serre +l	2,00	2,00	3,00	1,00	2800	9,00		2,00
	#NUL!	#NUL!	3,00	1,00	3000	9,00		1,00
valvulopatl	2,00	3,00	3,00	2,00	2600	7,00		1,00
RM serré +	2,00	33,00	3,00	1,00	3500	9,00		2,00
RM serre	2,00	3,00	6,00	2,00	2600	6,00		2,00
RM serre H	2,00	3,00	3,00	2,00	3000	9,00		1,00
SFA, rm se:	1,00	3,00	6,00	1,00	3100	8,00		1,00
rao serre b	1,00	3,00	4,00	2,00	3000	8,00	8 puis 9/1(	2,00
	#NUL!	#NUL!	5,00	2,00	2400	9,00		1,00

uterus bica	2,00	3,00	4,00	1,00	2400	3,00	3/10 puis 9	1,00
HTAP Rm s	2,00	3,00	5,00	2,00	2200	8,00		1,00
souffrance	2,00	3,00	6,00	1,00	2300	7,00		1,00
	#NUL!	#NUL!	3,00	2,00	3600	9,00		2,00
htap	2,00	2,00	3,00	1,00	2400	9,00		1,00
Rao serré s	2,00	3,00	5,00	2,00	2500	9,00		2,00
	#NUL!	#NUL!	#NUL!	#NUL!	#NUL!	#NUL!		#NUL!
RM serré H	2,00	3,00	3,00	2,00	2500	9,00		1,00
	2,00	1,00	3,00	1,00	2000	8,00		2,00
im importa	2,00	3,00	15,00	1,00	2200	8,00		1,00
rao serré s	2,00	3,00	3,00	2,00	3200	9,00		2,00

HOSP_CAR	cpcpostpar	TERME_HC	DECOMP_(	TERME_DE	ENDOCARI	TERME_EI	CPC_HEM(	TERME_HE
1,00	#NUL!	35,00	1,00	1,00	2,00	#NUL!	2,00	#NUL!
2,00	#NUL!	#NUL!	2,00	#NUL!	1,00	33,00	2,00	#NUL!
1,00	#NUL!	27,00	1,00	27,00	2,00	#NUL!	2,00	#NUL!
1,00	#NUL!	#NUL!	1,00	1,00	2,00	#NUL!	2,00	#NUL!
2,00	#NUL!	#NUL!	1,00	27,00	2,00	#NUL!	2,00	#NUL!
1,00	#NUL!	25,00	1,00	25,00	2,00	#NUL!	2,00	#NUL!
2,00	#NUL!	#NUL!	2,00	#NUL!	2,00	#NUL!	2,00	#NUL!
2,00	2,00	#NUL!	2,00	#NUL!	2,00	#NUL!	2,00	#NUL!
2,00	#NUL!	#NUL!	2,00	#NUL!	2,00	#NUL!	2,00	#NUL!
1,00	#NUL!	36,00	1,00	36,00	2,00	#NUL!	2,00	#NUL!
#NUL!	#NUL!	#NUL!	#NUL!	#NUL!	#NUL!	#NUL!	#NUL!	#NUL!
1,00	#NUL!	#NUL!	1,00	#NUL!	2,00	#NUL!	2,00	#NUL!
2,00	#NUL!	#NUL!	2,00	#NUL!	2,00	#NUL!	2,00	#NUL!
1,00	#NUL!	22,00	1,00	22,00	2,00	#NUL!	2,00	#NUL!
2,00	#NUL!	#NUL!	2,00	#NUL!	2,00	#NUL!	2,00	#NUL!
2,00	#NUL!	#NUL!	2,00	2,00	2,00	#NUL!	2,00	#NUL!
2,00	2,00	2,00	2,00	#NUL!	2,00	#NUL!	2,00	#NUL!
1,00	#NUL!	32,00	1,00	32,00	2,00	#NUL!	2,00	#NUL!
1,00	2,00	26,00	1,00	26,00	2,00	#NUL!	2,00	#NUL!
2,00	#NUL!	#NUL!	1,00	32,00	2,00	#NUL!	2,00	#NUL!
2,00	#NUL!	#NUL!	2,00	#NUL!	2,00	#NUL!	2,00	#NUL!
1,00	#NUL!	32,00	1,00	32,00	2,00	#NUL!	2,00	#NUL!
2,00	#NUL!	#NUL!	2,00	#NUL!	2,00	#NUL!	2,00	#NUL!
1,00	2,00	30,00	1,00	30,00	2,00	#NUL!	2,00	#NUL!
2,00	#NUL!	#NUL!	2,00	#NUL!	2,00	#NUL!	2,00	#NUL!
1,00	1,00	#NUL!	1,00	#NUL!	2,00	#NUL!	2,00	#NUL!
1,00	#NUL!	#NUL!	1,00	36,00	2,00	#NUL!	2,00	#NUL!
1,00	#NUL!	#NUL!	1,00	#NUL!	2,00	#NUL!	2,00	#NUL!
1,00	#NUL!	#NUL!	1,00	28,00	2,00	#NUL!	2,00	#NUL!
1,00	#NUL!	25,00	1,00	25,00	2,00	#NUL!	2,00	#NUL!
1,00	#NUL!	26,00	1,00	26,00	2,00	#NUL!	2,00	#NUL!
1,00	#NUL!	29,00	1,00	29,00	2,00	#NUL!	1,00	#NUL!
1,00	1,00	37,00	1,00	#NUL!	2,00	#NUL!	2,00	#NUL!
2,00	#NUL!	#NUL!	2,00	#NUL!	2,00	#NUL!	2,00	#NUL!
2,00	#NUL!	#NUL!	2,00	#NUL!	2,00	#NUL!	2,00	#NUL!
1,00	#NUL!	32,00	1,00	33,00	2,00	#NUL!	2,00	#NUL!
1,00	2,00	33,00	1,00	33,00	2,00	#NUL!	2,00	#NUL!
2,00	#NUL!	#NUL!	2,00	#NUL!	2,00	#NUL!	2,00	#NUL!
1,00	#NUL!	29,00	1,00	29,00	2,00	#NUL!	2,00	#NUL!
1,00	#NUL!	30,00	1,00	30,00	2,00	#NUL!	2,00	#NUL!
2,00	#NUL!	#NUL!	2,00	#NUL!	2,00	#NUL!	2,00	#NUL!
1,00	#NUL!	32,00	1,00	32,00	2,00	#NUL!	2,00	#NUL!
1,00	#NUL!	37,00	1,00	37,00	2,00	#NUL!	2,00	#NUL!
2,00	#NUL!	#NUL!	2,00	#NUL!	2,00	#NUL!	2,00	#NUL!
1,00	#NUL!	#NUL!	1,00	35,00	2,00	#NUL!	2,00	#NUL!
1,00	#NUL!	22,00	1,00	22,00	2,00	#NUL!	2,00	#NUL!
1,00	#NUL!	38,00	1,00	#NUL!	2,00	#NUL!	2,00	#NUL!
2,00	#NUL!	#NUL!	2,00	#NUL!	2,00	#NUL!	2,00	#NUL!
1,00	#NUL!	17,00	1,00	17,00	2,00	#NUL!	2,00	#NUL!

1,00	#NUL!	35,00	1,00	35,00	22,00	#NUL!	2,00	#NUL!
1,00	#NUL!	28,00	1,00	28,00	2,00	#NUL!	2,00	#NUL!
1,00	#NUL!	#NUL!	1,00	29,00	2,00	#NUL!	2,00	#NUL!
2,00	#NUL!	#NUL!	2,00	#NUL!	2,00	#NUL!	2,00	#NUL!
1,00	1,00	#NUL!	1,00	#NUL!	2,00	#NUL!	2,00	#NUL!
2,00	#NUL!	#NUL!	2,00	#NUL!	2,00	#NUL!	2,00	#NUL!
#NUL!	#NUL!	#NUL!	#NUL!	#NUL!	#NUL!	#NUL!	#NUL!	#NUL!
1,00	#NUL!	#NUL!	1,00	33,00	2,00	#NUL!	2,00	#NUL!
2,00	#NUL!	#NUL!	2,00	#NUL!	2,00	#NUL!	2,00	#NUL!
1,00	#NUL!	35,00	1,00	35,00	2,00	#NUL!	2,00	#NUL!
2,00	#NUL!	#NUL!	2,00	#NUL!	2,00	#NUL!	2,00	#NUL!

PASSAGE_I	TERME_FA	CPC_EMBC	TERME_Te	CPC_OBSTI	TOXEMIE1	DIABETEGE	MAP	RPM
2,00	#NUL!	2,00	#NUL!	2,00	2,00	2,00	2,00	2,00
2,00	#NUL!	2,00	#NUL!	1,00	2,00	2,00	1,00	2,00
2,00	#NUL!	2,00	#NUL!	2,00	2,00	2,00	2,00	2,00
2,00	#NUL!	2,00	#NUL!	2,00	2,00	2,00	2,00	2,00
2,00	#NUL!	2,00	#NUL!	1,00	2,00	2,00	1,00	2,00
1,00	#NUL!	2,00	#NUL!	2,00	2,00	2,00	2,00	2,00
2,00	#NUL!	2,00	#NUL!	2,00	2,00	2,00	2,00	2,00
2,00	#NUL!	2,00	#NUL!	2,00	2,00	2,00	2,00	2,00
2,00	#NUL!	2,00	#NUL!	2,00	2,00	2,00	2,00	2,00
2,00	#NUL!	2,00	#NUL!	2,00	2,00	2,00	2,00	2,00
#NUL!	#NUL!	#NUL!	#NUL!	#NUL!	#NUL!	#NUL!	#NUL!	#NUL!
2,00	#NUL!	2,00	#NUL!	2,00	2,00	2,00	1,00	2,00
2,00	#NUL!	2,00	#NUL!	2,00	2,00	2,00	2,00	2,00
2,00	#NUL!	2,00	#NUL!	2,00	2,00	2,00	2,00	2,00
2,00	#NUL!	2,00	#NUL!	2,00	2,00	2,00	2,00	2,00
2,00	#NUL!	2,00	#NUL!	2,00	2,00	2,00	2,00	2,00
2,00	#NUL!	2,00	#NUL!	2,00	2,00	2,00	2,00	2,00
2,00	#NUL!	2,00	#NUL!	2,00	2,00	2,00	2,00	2,00
1,00	26,00	2,00	#NUL!	1,00	2,00	2,00	1,00	2,00
2,00	#NUL!	2,00	#NUL!	1,00	2,00	2,00	1,00	2,00
2,00	#NUL!	2,00	#NUL!	2,00	2,00	2,00	2,00	2,00
2,00	#NUL!	2,00	#NUL!	2,00	2,00	2,00	2,00	2,00
2,00	#NUL!	2,00	#NUL!	2,00	2,00	2,00	2,00	2,00
2,00	#NUL!	2,00	#NUL!	2,00	2,00	2,00	2,00	2,00
2,00	#NUL!	2,00	#NUL!	2,00	2,00	2,00	2,00	2,00
2,00	#NUL!	1,00	#NUL!	2,00	2,00	2,00	2,00	2,00
2,00	#NUL!	2,00	#NUL!	2,00	2,00	2,00	2,00	2,00
2,00	#NUL!	2,00	#NUL!	2,00	2,00	2,00	2,00	2,00
2,00	#NUL!	2,00	#NUL!	2,00	2,00	2,00	2,00	2,00
2,00	#NUL!	2,00	#NUL!	2,00	2,00	2,00	2,00	2,00
2,00	#NUL!	2,00	#NUL!	2,00	2,00	2,00	2,00	2,00
2,00	#NUL!	2,00	#NUL!	1,00	1,00	2,00	1,00	2,00
2,00	#NUL!	2,00	#NUL!	2,00	2,00	2,00	2,00	2,00
2,00	#NUL!	2,00	#NUL!	2,00	2,00	2,00	2,00	2,00
2,00	#NUL!	2,00	#NUL!	2,00	2,00	2,00	2,00	2,00
2,00	#NUL!	2,00	#NUL!	1,00	2,00	2,00	2,00	2,00
1,00	#NUL!	2,00	#NUL!	1,00	2,00	2,00	1,00	1,00
2,00	#NUL!	2,00	#NUL!	1,00	2,00	2,00	1,00	2,00
2,00	#NUL!	2,00	#NUL!	1,00	1,00	2,00	2,00	2,00
2,00	#NUL!	2,00	#NUL!	2,00	2,00	2,00	2,00	2,00
2,00	#NUL!	2,00	#NUL!	1,00	1,00	2,00	2,00	2,00
2,00	#NUL!	2,00	#NUL!	1,00	2,00	2,00	1,00	2,00
2,00	#NUL!	2,00	#NUL!	2,00	2,00	2,00	2,00	2,00
2,00	#NUL!	2,00	#NUL!	1,00	2,00	1,00	2,00	2,00
2,00	#NUL!	2,00	#NUL!	1,00	2,00	2,00	1,00	2,00
1,00	22,00	2,00	#NUL!	2,00	2,00	2,00	2,00	2,00
2,00	#NUL!	2,00	#NUL!	2,00	2,00	2,00	2,00	2,00
2,00	#NUL!	2,00	#NUL!	2,00	2,00	2,00	2,00	2,00
1,00	17,00	2,00	#NUL!	2,00	2,00	2,00	2,00	2,00

2,00	#NUL!	2,00	#NUL!	1,00	2,00	2,00	1,00	2,00
2,00	#NUL!	2,00	#NUL!	2,00	2,00	2,00	2,00	2,00
2,00	#NUL!	2,00	#NUL!	1,00	2,00	2,00	1,00	2,00
2,00	#NUL!	2,00	#NUL!	2,00	2,00	2,00	2,00	2,00
2,00	#NUL!	2,00	#NUL!	2,00	2,00	2,00	2,00	2,00
2,00	#NUL!	2,00	#NUL!	1,00	2,00	2,00	1,00	2,00
#NUL!	#NUL!	#NUL!	#NUL!	#NUL!	#NUL!	#NUL!	#NUL!	#NUL!
2,00	#NUL!	2,00	#NUL!	2,00	2,00	2,00	2,00	2,00
2,00	#NUL!	2,00	#NUL!	2,00	2,00	2,00	2,00	2,00
2,00	#NUL!	2,00	#NUL!	1,00	2,00	2,00	1,00	2,00
2,00	#NUL!	2,00	#NUL!	2,00	2,00	2,00	2,00	2,00

HGIE_PERI	hGIE_POST	DECES_MA	CAUSE_DE	CPC_FOET	MFIU	HYPOTROF	PREMATU	MORTALIT
2,00	2,00	2,00	#NUL!	2,00	2,00	2,00	2,00	2,00
2,00	2,00	2,00	#NUL!	2,00	2,00	2,00	2,00	2,00
2,00	2,00	2,00	#NUL!	2,00	2,00	2,00	2,00	2,00
2,00	1,00	2,00	#NUL!	2,00	2,00	2,00	2,00	2,00
2,00	2,00	2,00	#NUL!	1,00	2,00	1,00	1,00	2,00
2,00	2,00	2,00	#NUL!	2,00	2,00	2,00	2,00	2,00
2,00	2,00	2,00	#NUL!	2,00	2,00	2,00	2,00	2,00
2,00	2,00	2,00	#NUL!	2,00	2,00	2,00	2,00	2,00
2,00	2,00	2,00	#NUL!	2,00	2,00	2,00	2,00	2,00
2,00	2,00	2,00	#NUL!	2,00	2,00	2,00	2,00	2,00
#NUL!	#NUL!	#NUL!	#NUL!	#NUL!	#NUL!	#NUL!	#NUL!	#NUL!
2,00	2,00	2,00	#NUL!	1,00	2,00	2,00	1,00	2,00
2,00	2,00	2,00	#NUL!	2,00	2,00	2,00	2,00	2,00
2,00	2,00	2,00	#NUL!	2,00	2,00	2,00	2,00	2,00
2,00	2,00	2,00	#NUL!	2,00	2,00	2,00	2,00	2,00
2,00	2,00	2,00	#NUL!	1,00	2,00	2,00	2,00	2,00
2,00	2,00	2,00	#NUL!	2,00	2,00	2,00	2,00	2,00
2,00	2,00	2,00	#NUL!	2,00	2,00	2,00	2,00	2,00
2,00	2,00	2,00	#NUL!	1,00	2,00	1,00	2,00	2,00
2,00	2,00	2,00	#NUL!	1,00	2,00	1,00	1,00	2,00
2,00	2,00	2,00	#NUL!	2,00	2,00	2,00	2,00	2,00
2,00	2,00	2,00	#NUL!	1,00	2,00	1,00	2,00	2,00
2,00	2,00	2,00	#NUL!	2,00	2,00	2,00	2,00	2,00
2,00	2,00	2,00	#NUL!	2,00	2,00	2,00	2,00	2,00
2,00	2,00	2,00	#NUL!	2,00	2,00	2,00	2,00	2,00
2,00	2,00	2,00	#NUL!	2,00	2,00	2,00	2,00	2,00
2,00	2,00	2,00	#NUL!	1,00	2,00	1,00	2,00	2,00
2,00	2,00	2,00	#NUL!	1,00	2,00	1,00	2,00	2,00
2,00	2,00	2,00	#NUL!	2,00	2,00	2,00	2,00	2,00
2,00	2,00	2,00	#NUL!	2,00	2,00	2,00	2,00	2,00
2,00	2,00	2,00	#NUL!	2,00	2,00	2,00	2,00	2,00
2,00	2,00	2,00	#NUL!	1,00	2,00	2,00	1,00	2,00
2,00	2,00	2,00	#NUL!	2,00	2,00	2,00	1,00	2,00
2,00	2,00	2,00	#NUL!	2,00	2,00	2,00	2,00	2,00
2,00	2,00	2,00	#NUL!	2,00	2,00	2,00	2,00	2,00
2,00	2,00	2,00	#NUL!	1,00	2,00	1,00	2,00	2,00
2,00	2,00	2,00	#NUL!	1,00	2,00	1,00	1,00	2,00
2,00	2,00	2,00	#NUL!	2,00	2,00	2,00	1,00	2,00
2,00	2,00	2,00	#NUL!	1,00	2,00	1,00	2,00	2,00
2,00	2,00	2,00	#NUL!	2,00	2,00	2,00	2,00	2,00
2,00	2,00	2,00	#NUL!	2,00	2,00	2,00	2,00	2,00
2,00	2,00	2,00	#NUL!	2,00	2,00	2,00	2,00	2,00
2,00	2,00	2,00	#NUL!	1,00	2,00	1,00	2,00	2,00
2,00	2,00	2,00	#NUL!	2,00	2,00	2,00	2,00	2,00
2,00	2,00	2,00	#NUL!	1,00	2,00	2,00	1,00	2,00
2,00	2,00	2,00	#NUL!	2,00	2,00	2,00	2,00	2,00
2,00	2,00	2,00	#NUL!	2,00	2,00	2,00	2,00	2,00
2,00	2,00	2,00	#NUL!	2,00	2,00	2,00	2,00	2,00
2,00	2,00	2,00	#NUL!	1,00	2,00	1,00	2,00	2,00

2,00	2,00	2,00	#NUL!	1,00	2,00	2,00	1,00	2,00
2,00	2,00	2,00	#NUL!	1,00	2,00	1,00	1,00	2,00
2,00	2,00	2,00	#NUL!	1,00	2,00	1,00	1,00	2,00
2,00	2,00	2,00	#NUL!	2,00	2,00	2,00	2,00	2,00
2,00	2,00	2,00	#NUL!	1,00	2,00	1,00	2,00	2,00
2,00	2,00	2,00	#NUL!	1,00	2,00	1,00	2,00	2,00
#NUL!	#NUL!	#NUL!	#NUL!	#NUL!	#NUL!	#NUL!	#NUL!	#NUL!
2,00	2,00	2,00	#NUL!	1,00	2,00	1,00	2,00	2,00
2,00	2,00	2,00	#NUL!	1,00	2,00	1,00	2,00	2,00
2,00	2,00	2,00	#NUL!	1,00	2,00	2,00	1,00	2,00
2,00	2,00	2,00	#NUL!	2,00	2,00	2,00	2,00	2,00

HOSPENNE	CAUSE1	DMPCPENI	CI_dmpc	TERME_DN	SM_AVT	SM_APRES	CPC_DMP(	ARRET_AV
2,00	#NUL!	2,00		#NUL!	#NUL!	#NUL!	#NUL!	#NUL!
2,00	#NUL!	#NUL!		#NUL!	#NUL!	#NUL!	#NUL!	1,00
2,00	#NUL!	#NUL!		#NUL!	#NUL!	#NUL!	#NUL!	#NUL!
2,00	#NUL!	2,00	calcication	#NUL!	#NUL!	#NUL!	#NUL!	1,00
1,00	#NUL!	1,00		29,00	1,20	1,50	1,00	#NUL!
2,00	#NUL!	2,00		#NUL!	#NUL!	#NUL!	#NUL!	1,00
2,00	#NUL!	#NUL!		#NUL!	#NUL!	#NUL!	#NUL!	#NUL!
2,00	#NUL!	2,00		#NUL!	#NUL!	#NUL!	#NUL!	#NUL!
2,00	#NUL!	#NUL!		#NUL!	#NUL!	#NUL!	#NUL!	#NUL!
2,00	#NUL!	2,00		#NUL!	#NUL!	#NUL!	#NUL!	#NUL!
#NUL!	#NUL!	#NUL!		#NUL!	#NUL!	#NUL!	#NUL!	#NUL!
2,00	#NUL!	2,00	im	#NUL!	#NUL!	#NUL!	#NUL!	#NUL!
2,00	#NUL!	#NUL!		#NUL!	#NUL!	#NUL!	#NUL!	#NUL!
2,00	#NUL!	2,00		#NUL!	#NUL!	#NUL!	#NUL!	#NUL!
2,00	#NUL!	2,00		#NUL!	#NUL!	#NUL!	#NUL!	#NUL!
1,00	#NUL!	2,00		#NUL!	#NUL!	#NUL!	#NUL!	#NUL!
2,00	#NUL!	#NUL!		#NUL!	#NUL!	#NUL!	#NUL!	#NUL!
2,00	#NUL!	1,00		34,00	1,20	1,80	2,00	2,00
2,00	#NUL!	1,00		27,00	1,30	1,60	#NUL!	#NUL!
1,00	#NUL!	2,00	rm entonn	#NUL!	#NUL!	#NUL!	#NUL!	#NUL!
2,00	#NUL!	2,00		#NUL!	#NUL!	#NUL!	#NUL!	#NUL!
2,00	#NUL!	2,00	thrombus (	#NUL!	#NUL!	#NUL!	#NUL!	1,00
2,00	#NUL!	2,00		#NUL!	#NUL!	#NUL!	#NUL!	1,00
2,00	#NUL!	1,00		30,00	1,20	1,80	2,00	#NUL!
2,00	#NUL!	2,00	rm entonn	#NUL!	#NUL!	#NUL!	#NUL!	#NUL!
2,00	#NUL!	#NUL!		#NUL!	#NUL!	#NUL!	#NUL!	#NUL!
2,00	#NUL!	2,00	im importa	#NUL!	#NUL!	#NUL!	#NUL!	#NUL!
2,00	#NUL!	2,00		#NUL!	#NUL!	#NUL!	#NUL!	#NUL!
2,00	#NUL!	2,00		#NUL!	#NUL!	#NUL!	#NUL!	#NUL!
2,00	#NUL!	1,00		#NUL!	1,10	1,80	2,00	1,00
2,00	#NUL!	1,00		27,00	1,00	1,20	2,00	#NUL!
2,00	#NUL!	2,00	IM garde II	#NUL!	#NUL!	#NUL!	#NUL!	1,00
2,00	#NUL!	#NUL!		#NUL!	#NUL!	#NUL!	#NUL!	#NUL!
2,00	#NUL!	2,00		#NUL!	#NUL!	#NUL!	#NUL!	#NUL!
2,00	#NUL!	1,00		29,00	1,00	1,80	2,00	#NUL!
2,00	#NUL!	2,00	im	#NUL!	#NUL!	#NUL!	#NUL!	#NUL!
2,00	#NUL!	#NUL!		#NUL!	#NUL!	#NUL!	#NUL!	#NUL!
2,00	#NUL!	2,00	im	#NUL!	#NUL!	#NUL!	#NUL!	#NUL!
2,00	#NUL!	2,00		#NUL!	#NUL!	#NUL!	#NUL!	1,00
2,00	#NUL!	2,00	rm calcifie	#NUL!	#NUL!	#NUL!	#NUL!	1,00
2,00	#NUL!	2,00	im	#NUL!	#NUL!	#NUL!	#NUL!	1,00
2,00	#NUL!	1,00		32,00	1,20	1,60	2,00	#NUL!
2,00	#NUL!	2,00		#NUL!	#NUL!	#NUL!	#NUL!	#NUL!
2,00	#NUL!	2,00	entonnoir	#NUL!	#NUL!	#NUL!	#NUL!	#NUL!
2,00	#NUL!	2,00	calcifie	#NUL!	#NUL!	#NUL!	#NUL!	2,00
2,00	#NUL!	#NUL!		#NUL!	#NUL!	#NUL!	#NUL!	1,00
2,00	#NUL!	2,00		2,00	#NUL!	#NUL!	#NUL!	#NUL!
2,00	#NUL!	#NUL!		#NUL!	#NUL!	#NUL!	#NUL!	#NUL!
2,00	#NUL!	1,00		20,00	0,97	2,10	2,00	2,00

2,00	#NUL!	2,00		#NUL!	#NUL!	#NUL!	#NUL!	1,00
2,00	#NUL!	2,00		#NUL!	#NUL!	#NUL!	#NUL!	#NUL!
1,00	#NUL!	2,00		#NUL!	#NUL!	#NUL!	#NUL!	#NUL!
2,00	#NUL!	#NUL!		#NUL!	#NUL!	#NUL!	#NUL!	#NUL!
2,00	#NUL!	2,00	calcificatio	#NUL!	#NUL!	#NUL!	#NUL!	#NUL!
1,00	#NUL!	2,00		#NUL!	#NUL!	#NUL!	#NUL!	1,00
#NUL!	#NUL!	#NUL!		#NUL!	#NUL!	#NUL!	#NUL!	#NUL!
2,00	#NUL!	2,00		#NUL!	#NUL!	#NUL!	#NUL!	#NUL!
2,00	#NUL!	2,00		#NUL!	#NUL!	#NUL!	#NUL!	#NUL!
2,00	#NUL!	2,00		#NUL!	#NUL!	#NUL!	#NUL!	2,00
2,00	#NUL!	#NUL!		#NUL!	#NUL!	#NUL!	#NUL!	#NUL!

HOSP_CAR	SWITCH_H	SWITCH_H	RPRISE_AV	TRANSFER	GESTION_A	ALLAITEME	CONTRACE	commenta
#NUL!	#NUL!	#NUL!	#NUL!	#NUL!	#NUL!	2,00	2,00	
1,00	1,00	2,00	4,00	2,00	1,00	2,00	1,00	
#NUL!	#NUL!	#NUL!	#NUL!	#NUL!	#NUL!	1,00	2,00	
1,00	1,00	2,00	15,00	1,00	2,00	1,00	1,00	ligature de
#NUL!	#NUL!	#NUL!	#NUL!	#NUL!	#NUL!	1,00	2,00	arrest card
2,00	2,00	1,00	4,00	2,00	1,00	2,00	#NUL!	
#NUL!	#NUL!	#NUL!	#NUL!	#NUL!	#NUL!	1,00	1,00	
#NUL!	#NUL!	#NUL!	#NUL!	#NUL!	#NUL!	1,00	1,00	calendrier
#NUL!	#NUL!	#NUL!	#NUL!	#NUL!	#NUL!	1,00	1,00	LIGATURE
#NUL!	#NUL!	#NUL!	#NUL!	#NUL!	#NUL!	1,00	#NUL!	
#NUL!	#NUL!	#NUL!	#NUL!	#NUL!	#NUL!	#NUL!	#NUL!	
#NUL!	#NUL!	#NUL!	#NUL!	#NUL!	#NUL!	#NUL!	#NUL!	
#NUL!	#NUL!	#NUL!	#NUL!	#NUL!	#NUL!	2,00	2,00	
2,00	#NUL!	#NUL!	#NUL!	#NUL!	#NUL!	1,00	1,00	microproge
#NUL!	#NUL!	#NUL!	#NUL!	#NUL!	#NUL!	1,00	1,00	
2,00	2,00	2,00	#NUL!	2,00	#NUL!	1,00	1,00	refuse ligat
#NUL!	#NUL!	#NUL!	#NUL!	#NUL!	#NUL!	1,00	2,00	
#NUL!	#NUL!	#NUL!	#NUL!	#NUL!	#NUL!	1,00	2,00	
2,00	1,00	2,00	5,00	2,00	1,00	1,00	2,00	
#NUL!	#NUL!	#NUL!	#NUL!	#NUL!	#NUL!	1,00	1,00	
#NUL!	#NUL!	#NUL!	#NUL!	#NUL!	#NUL!	1,00	1,00	preservatif
2,00	2,00	1,00	4,00	2,00	1,00	1,00	#NUL!	
1,00	2,00	1,00	4,00	2,00	1,00	1,00	2,00	
#NUL!	#NUL!	#NUL!	#NUL!	#NUL!	#NUL!	1,00	2,00	
#NUL!	#NUL!	#NUL!	#NUL!	#NUL!	#NUL!	1,00	1,00	
#NUL!	#NUL!	#NUL!	#NUL!	#NUL!	#NUL!	1,00	1,00	
#NUL!	#NUL!	#NUL!	#NUL!	#NUL!	#NUL!	1,00	2,00	
#NUL!	#NUL!	#NUL!	#NUL!	#NUL!	#NUL!	1,00	1,00	Meadows
#NUL!	#NUL!	#NUL!	#NUL!	#NUL!	#NUL!	1,00	1,00	
1,00	2,00	1,00	4,00	2,00	1,00	1,00	1,00	
#NUL!	#NUL!	#NUL!	#NUL!	#NUL!	#NUL!	1,00	2,00	
1,00	1,00	2,00	3,00	2,00	1,00	2,00	1,00	
#NUL!	#NUL!	#NUL!	#NUL!	#NUL!	#NUL!	1,00	1,00	
#NUL!	#NUL!	#NUL!	#NUL!	#NUL!	#NUL!	1,00	1,00	
#NUL!	#NUL!	#NUL!	#NUL!	#NUL!	#NUL!	1,00	1,00	
2,00	1,00	2,00	5,00	2,00	1,00	1,00	2,00	
#NUL!	#NUL!	1,00	5,00	1,00	2,00	2,00	1,00	
#NUL!	#NUL!	#NUL!	#NUL!	#NUL!	#NUL!	1,00	2,00	
2,00	2,00	1,00	3,00	2,00	1,00	2,00	2,00	auccune
1,00	2,00	1,00	4,00	2,00	1,00	2,00	2,00	
2,00	2,00	1,00	3,00	2,00	1,00	2,00	2,00	
#NUL!	#NUL!	#NUL!	#NUL!	#NUL!	#NUL!	1,00	2,00	
2,00	#NUL!	#NUL!	#NUL!	2,00	#NUL!	1,00	2,00	
#NUL!	#NUL!	#NUL!	#NUL!	#NUL!	#NUL!	1,00	1,00	
2,00	#NUL!	#NUL!	#NUL!	1,00	2,00	1,00	1,00	
2,00	#NUL!	2,00	3,00	2,00	1,00	1,00	1,00	metroraag
2,00	#NUL!	#NUL!	#NUL!	#NUL!	2,00	1,00	2,00	
2,00	#NUL!	#NUL!	#NUL!	#NUL!	#NUL!	1,00	1,00	
#NUL!	#NUL!	#NUL!	#NUL!	#NUL!	#NUL!	1,00	1,00	

1,00	1,00	2,00	4,00	2,00	1,00	1,00	2,00	
#NUL!	#NUL!	#NUL!	#NUL!	#NUL!	#NUL!	1,00	1,00	ligature
#NUL!	#NUL!	#NUL!	#NUL!	#NUL!	#NUL!	2,00	1,00	
#NUL!	#NUL!	#NUL!	#NUL!	#NUL!	#NUL!	1,00	2,00	
#NUL!	#NUL!	#NUL!	#NUL!	#NUL!	#NUL!	1,00	2,00	
1,00	1,00	2,00	4,00	2,00	1,00	1,00	2,00	Rao sous va
#NUL!	#NUL!	#NUL!	#NUL!	#NUL!	#NUL!	#NUL!	#NUL!	
#NUL!	#NUL!	#NUL!	#NUL!	#NUL!	#NUL!	1,00	1,00	
2,00	#NUL!	#NUL!	#NUL!	#NUL!	#NUL!	1,00	1,00	
2,00	2,00	2,00	2,00	1,00	2,00	1,00	1,00	
#NUL!	#NUL!	#NUL!	#NUL!	#NUL!	#NUL!	1,00	2,00	

moy_contr	DMPC_apr	RVM_apre	delaidmpc_	VAR00003
	#NUL!	#NUL!	#NUL!	
ligature de	#NUL!	1,00	15,00	
	#NUL!	#NUL!	#NUL!	
s trompes	2,00	2,00	#NUL!	thrombopenie splenomegalie transufusio 10cp oap en p
iaque	#NUL!	#NUL!	#NUL!	
	#NUL!	#NUL!	#NUL!	
ligature trc	#NUL!	1,00	#NUL!	ATCD meadows , en 2014, avec dysfonction VG
	#NUL!	#NUL!	#NUL!	cornarienne, dyslipidémie familiale, plastie du TCG a l'ag
LIGATURE	2,00	1,00	8,00	
	#NUL!	#NUL!	#NUL!	
	#NUL!	#NUL!	#NUL!	
	#NUL!	#NUL!	#NUL!	
	#NUL!	#NUL!	#NUL!	
estatit	#NUL!	#NUL!	#NUL!	
ligature	#NUL!	#NUL!	#NUL!	
ture trompe	#NUL!	#NUL!	#NUL!	
	#NUL!	#NUL!	#NUL!	dilatation pulmonaire pour RP serré valvulaire, Gmax 8
auccun	#NUL!	#NUL!	#NUL!	
	#NUL!	#NUL!	#NUL!	tombé enceinte apres deux ans (2017, avec RM serré)
ligature trc	2,00	1,00	8,00	
	2,00	2,00	#NUL!	
	1,00	#NUL!	7,00	
	2,00	2,00	#NUL!	
auccun	#NUL!	#NUL!	#NUL!	
microprog	#NUL!	#NUL!	#NUL!	
auccun	#NUL!	#NUL!	#NUL!	
	#NUL!	#NUL!	#NUL!	
sterilet	1,00	#NUL!	12,00	
calendrier	1,00	#NUL!	9,00	
ligature	#NUL!	#NUL!	#NUL!	
	#NUL!	#NUL!	#NUL!	
ligature de	2,00	1,00	12,00	
microprog	#NUL!	#NUL!	#NUL!	
calendrier	2,00	2,00	#NUL!	
calendrier	#NUL!	#NUL!	#NUL!	
	2,00	1,00	9,00	
calendrier	1,00	#NUL!	#NUL!	le jour de la DMPc, RPM souffrance foetale , cesarienne
auccun	#NUL!	#NUL!	#NUL!	
auccune	2,00	1,00	13,00	
auccun	2,00	1,00	12,00	
	#NUL!	#NUL!	#NUL!	
	#NUL!	#NUL!	#NUL!	
	#NUL!	#NUL!	#NUL!	
progetstati	#NUL!	1,00	15,00	
progestatif	2,00	1,00	15,00	
diu/ligatur	2,00	2,00	#NUL!	
	#NUL!	#NUL!	#NUL!	
progestatif	#NUL!	#NUL!	#NUL!	
ligature trc	#NUL!	#NUL!	#NUL!	

	#NUL!	#NUL!	#NUL!
ligature	2,00	1,00	6,00
ligature trc	#NUL!	#NUL!	#NUL!
auccun	#NUL!	1,00	#NUL!
	#NUL!	#NUL!	#NUL!
alvulaire	#NUL!	#NUL!	#NUL!
	#NUL!	#NUL!	#NUL!
calednrier	#NUL!	#NUL!	#NUL!
calendrier	1,00	#NUL!	12,00
ligature trc	#NUL!	#NUL!	#NUL!
	#NUL!	1,00	12,00

›ostcesarienne transferée en reanimation , intubé ventil
